# Light-regulated dual-targeting of NUCLEAR CONTROL OF PEP ACTIVITY establishes photomorphogenesis via interorganellar communication

**DOI:** 10.1093/plphys/kiaf289

**Published:** 2025-06-30

**Authors:** Jae-Hyung Lee, Thu Minh Doan, Abigail Bruzual, Sandhya Senthilkumar, Chan Yul Yoo

**Affiliations:** School of Biological Sciences, University of Utah, Salt Lake City, UT 84112, USA; School of Biological Sciences, University of Utah, Salt Lake City, UT 84112, USA; School of Biological Sciences, University of Utah, Salt Lake City, UT 84112, USA; School of Biological Sciences, University of Utah, Salt Lake City, UT 84112, USA; School of Biological Sciences, University of Utah, Salt Lake City, UT 84112, USA

## Abstract

Interorganellar communication is essential for maintaining cellular and organellar functions and adapting to dynamic environmental changes in eukaryotic cells. In angiosperms, light initiates photomorphogenesis, a developmental program characterized by chloroplast biogenesis and inhibition of hypocotyl elongation, through photoreceptors such as the red-/far-red-sensing phytochromes and their downstream signaling pathways. However, the mechanisms underlying nucleus–chloroplast crosstalk during photomorphogenesis remain elusive. Here, we show that light-regulated dual-targeting of NUCLEAR CONTROL OF PEP ACTIVITY (NCP) mediates bidirectional communication between the nucleus and chloroplasts via alternative promoter selection and retrograde translocation. Light promotes transcription from an upstream canonical transcription start site, producing a long NCP (NCP-L) isoform containing an N-terminal chloroplast transit peptide that directs chloroplast localization. In contrast, darkness or low red light conditions favor transcription from a downstream alternative start site, producing a shorter cytoplasmic isoform (NCP-S) that is rapidly degraded via the 26S proteasome. This light-regulated alternative transcription initiation depends on PHYTOCHROME-INTERACTING FACTORS (PIFs), key repressors of photomorphogenesis. Upon chloroplast import, NCP-L is processed into its mature form (NCPm), which promotes assembly and nucleoid localization of the plastid-encoded RNA polymerase (PEP) complex to initiate chloroplast biogenesis. Notably, NCP's nuclear function requires its prior localization to chloroplasts, supporting a model in which NCP mediates chloroplast-to-nucleus retrograde signaling. Consistent with this, NCP promotes stromule formation in Arabidopsis (*Arabidopsis thaliana*) hypocotyls, linking chloroplast dynamics to phytochrome-dependent nuclear pathways that restrict hypocotyl elongation. Our findings uncover an interorganellar communication mechanism in which light-dependent alternative promoter usage and retrotranslocation regulate photomorphogenesis, integrating nuclear and plastid signals to coordinate organ-specific developmental programs.

## Teaser

Light regulates communication between the nucleus and chloroplasts to coordinate plant development and organelle function during photomorphogenesis.

## Introduction

The eukaryotic cell is distinguished by its membrane-bound organelles, including the nucleus, endoplasmic reticulum, vacuoles, Golgi apparatus, and mitochondria. In plants and algae, chloroplasts are essential organelles that evolved from a free-living cyanobacterial ancestor through endosymbiosis and are responsible for photosynthesis (Archibald 2015). Chloroplasts are semiautonomous, as most genes in the plastidial genome have been either lost or transferred to the nuclear genome during the coevolution of plastids and their host cells (Jarvis and López-Juez 2013). However, the plastidial genome still retains key genes encoding core components of the PEP, the translation machinery, and the photosystems, which are essential for chloroplast development and function. This dual genetic control necessitates the coordination of gene expression from both the nuclear and plastidial genomes to regulate chloroplast biogenesis, function, and homeostasis (Woodson and Chory 2008). Furthermore, chloroplasts communicate bidirectionally with the nucleus in response to internal and external stimuli to regulate nuclear gene expression (Wang et al. 2020). In this interorganellar communication, anterograde signaling from the nucleus to chloroplasts refers to the control of chloroplast development and function by nuclear-encoded factors, while retrograde signaling conveys information from chloroplasts back to the nucleus to modulate gene expression accordingly (Woodson and Chory 2008; Griffin and Toledo-Ortiz 2022). This bidirectional communication is essential for orchestrating plant growth and development, as well as facilitating adaptation to environmental changes (Griffin and Toledo-Ortiz 2022).

Light is an essential environmental cue for plants and, particularly in angiosperms, initiates developmental programs involving chloroplast biogenesis (Waters and Langdale 2009). When seeds germinate in the dark, a developmental program called skotomorphogenesis promotes hypocotyl elongation, allowing seedlings to emerge from the soil. This process inhibits chloroplast biogenesis to prevent photooxidative damage when seedlings encounter light later. Upon light exposure, a developmental program called photomorphogenesis is activated to restrict hypocotyl growth and initiate the differentiation of plastids into photosynthetically active chloroplasts (Chen and Chory 2011). Hypocotyl growth is controlled by genome-wide transcriptional changes in the nucleus (Shi et al. 2018). Chloroplast biogenesis requires the synthesis of plastid-localized proteins to generate functional photosynthetic components such as the light-harvesting chlorophyll a/b complex, photosystems, ribulose bisphosphate carboxylase/oxygenase, and thylakoid membranes where photosynthetic machineries are built. These proteins are encoded by photosynthesis-associated nuclear genes (*PhANGs*) and plastid genes (*PhAPGs*), which are transcribed from both nuclear and plastidial genomes (Woodson and Chory 2008; Yoo et al. 2020). Establishing photomorphogenesis, therefore, requires the coordinated gene expression from the nuclear and plastidial genomes (Yoo et al. 2020).

Light is perceived by a suite of photoreceptors, including the red- and far-red-light-sensing phytochromes (PHYs) (Chen et al. 2004). In the dark, inactive PHYs are localized in the cytoplasm, but upon light activation, PHYs translocate into the nucleus and form condensates called photobodies (Chen et al. 2004; Willige et al. 2024). PHY signaling reprograms nuclear gene expression by regulating the stability and activity of PHYTOCHROME-INTERACTING FACTORS (PIFs), which are master repressors of photomorphogenesis (Pham et al. 2018; Cheng et al. 2021). In the dark, most PIFs accumulate and activate growth-related genes in the nucleus, promoting hypocotyl growth. PIFs also repress nuclear and plastidial genes associated with photosynthesis and chloroplast biogenesis (Yoo et al. 2019; Hwang et al. 2022). Thus, photoactivated PHYs repress PIF activity and stability in the nucleus, triggering nucleus-to-plastid anterograde signaling to activate plastid gene expression (Yoo et al. 2020).

Plastid genes are transcribed by the plastid-encoded RNA polymerase (PEP) and the nuclear-encoded RNA polymerase (NEP) (Börner et al. 2015). PEP, which originated in bacteria, is a multisubunit RNA polymerase complex consisting of 4 bacterial-like core subunits and at least 15 plant-specific PEP-associated proteins (PAPs). The bacterial-like core subunits are plastid genes (*rpoA*, *rpoB*, *rpoC1*, and *rpoC2*) transcribed by NEP, a phage-type RNA polymerase. The PAPs, encoded by nuclear genes, are imported into chloroplasts and assembled with the core subunits to form a functional PEP holoenzyme complex. Recent cryo-electron microscopy studies in tobacco (*Nicotiana tabacum*) and white mustard reveal that PAPs are both structural components of the PEP complex and exhibit other enzymatic activities associated with PEP function in chloroplasts (do Prado et al. 2024; Vergara-Cruces et al. 2024; Wu et al. 2024). PEP assembly is triggered by light via PHY signaling and is repressed in darkness by PIFs in the nucleus (Yoo et al. 2019). In addition, PEP activity can influence PHY-regulated nuclear gene expression, as well as nuclear-encoded photosynthesis genes, through retrograde signaling (Koussevitzky et al. 2007; Woodson et al. 2013; Martín et al. 2016). These characteristics suggest that nucleus–chloroplast interorganellar communication is crucial for establishing and maintaining photomorphogenesis.

A forward genetic screen aimed at identifying PHY signaling components for chloroplast biogenesis led to the discovery of REGULATOR OF CHLOROPLAST BIOGENESIS (RCB) and NUCLEAR CONTROL OF PEP ACTIVITY (NCP) in Arabidopsis (*Arabidopsis thaliana*) (Yang et al. 2019; Yoo et al. 2019). RCB and NCP are dual-localized to both the nucleus and plastids, where they play roles in 2 major photomorphogenic developmental processes: hypocotyl growth inhibition and chloroplast biogenesis. In the nucleus, both RCB and NCP are required for the formation of large phyB photobodies and the degradation of PIF1 and PIF3 to inhibit hypocotyl elongation. PHY-mediated PIF degradation in the nucleus sends anterograde signals to plastids, activating *PhAPGs* expression by triggering PEP assembly and activating sigma factors for promoter recognition (Yoo et al. 2019; Hwang et al. 2022). RCB activates PEP assembly primarily in the nucleus by degrading PIFs (Yoo et al. 2019). Notably, NCP plays pivotal dual roles in controlling PIF degradation in the nucleus and PEP assembly in plastids (Yang et al. 2019). These genetic studies laid the foundation for understanding nucleus–chloroplast communication (Yoo et al. 2020). However, the nucleus–chloroplast interorganellar communication mechanisms governing the dual localization of these proteins and their regulation by light remain to be understood. In particular, the dual localization of RCB and NCP raises the question of how light controls their localization to coordinate hypocotyl growth inhibition in the nucleus and chloroplast biogenesis in the plastids.

In plants, PHYs induce genome-wide changes in light-dependent alternative splicing and alternative promoter selection, generating distinct transcript variants and protein isoforms with diverse functions and subcellular localizations (Shikata et al. 2014; Ushijima et al. 2017). This prompted us to investigate whether the dual localization of RCB and NCP is regulated at the transcript level via alternative promoter usage. In this study, we found that light regulates the alternative transcription initiation of *NCP*, but not *RCB*, generating a long NCP (NCP-L) isoform with an N-terminal chloroplast transit peptide and a short isoform lacking this N-terminal sequence. While the short NCP isoform is degraded in the cytoplasm by the 26S proteasome, the NCP-L isoform primarily localizes to chloroplasts to initiate chloroplast biogenesis and subsequently translocates to the nucleus, potentially via stromules, to inhibit hypocotyl elongation. Our results demonstrate a dynamic regulatory mechanism via alternative promoter usage and retrotranslocation to mediate interorganellar communication. This study reveals a molecular framework for bidirectional signaling between the nucleus and plastids that coordinates chloroplast biogenesis and hypocotyl elongation during photomorphogenesis.

## Results

### Light regulates the alternative transcription initiation of *NCP* in a PIF-dependent manner

The nucleus- and chloroplast-localized NCP and RCB paralogs contain a chloroplast-targeting transit peptide (cTP) in their N termini and a predicted nuclear localization signal (NLS) in the middle region (Yang et al. 2019; Yoo et al. 2019). We hypothesized that PHY-mediated alternative promoter selection could generate different NCP or RCB isoforms, either containing or lacking the cTP (Ushijima et al. 2017). To test this, we performed 5′ rapid amplification of cDNA ends (5′ RACE) analysis on total RNA extracted from seedlings grown under white light (WL) and darkness. While a single *NCP* transcript was detected in seedlings grown under WL, 2 *NCP* transcript variants were detected in the dark condition (Supplementary Fig. S1, A and B). Sequencing the longer *NCP* cDNA (*NCP-L*) and the shorter *NCP* cDNA (*NCP-S*) revealed that the *NCP-L* transcript initiates within the transcription start sites (TSSs) annotated in The Arabidopsis Information Resource (referred to here as TSS1) (Supplementary Figs. S1B and S2A). The *NCP-S* transcript, however, initiates from TSS2, located after the first ATG in the coding sequence (Supplementary Fig. S2A), resulting in the production of a short isoform with a truncated N terminus (Supplementary Fig. S3). In contrast, 5′ RACE analysis of the *RCB* transcript showed a single transcript under both WL and dark conditions (Supplementary Figs. S1, C and D and S2B). These results indicate that 2 *NCP* transcript variants, with different TSSs, are present in the dark but not in the light.

**Figure 1.**
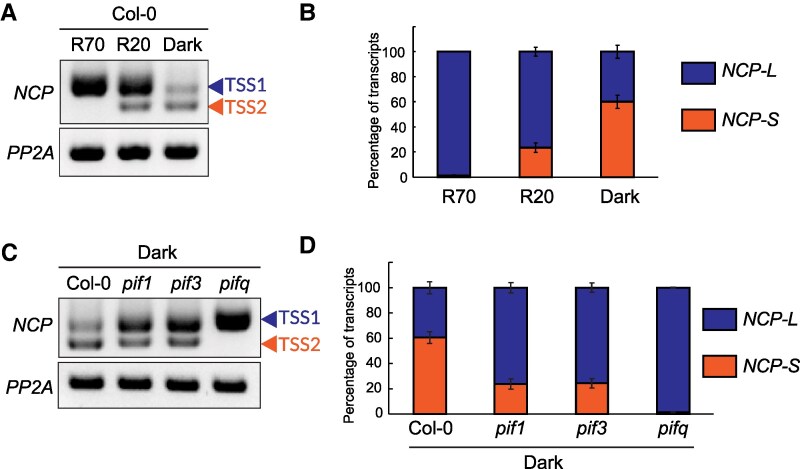
Light regulates alternative TSSs in *NCP* via a PIF-dependent manner. **A)** Effect of light intensity on use of *NCP* alternative promoters. Col-0 plants were grown for 4 d under 70 *μ*mol m^−2^ s^−1^ red light (R70), 20 *μ*mol m^−2^ s^−1^ red light (R20), or true-dark conditions for 4 d before extracting total RNA from whole seedlings. The TSSs by 5′ RACE analysis were designated TSS1 (upper arrowhead) and TSS2 (lower arrowhead). *PP2A* was used as an internal control. **B)** Quantification of longer and shorter *NCP* transcripts. Quantification was performed using 5′ RACE-PCR in **A)**. DNA blots on the 3 5′ RACE-PCR from biological triplicate samples were used for quantification. Blue and orange boxes indicate percentage of quantified band for the longer (*NCP-L*) and shorter (*NCP-S*) *NCP* transcripts, respectively. Error bars indicate Sd of the 3 biological replicates. **C)** Effect of PIF mutations on the alternative promoter usage in *NCP*. Col-0, *pif1*, *pif3*, and *pifq* plants were grown for 4 d under true-dark conditions before extracting total RNA from whole seedlings. Each transcription start site identified by 5′ RACE-PCR was marked as TSS1 (blue arrowhead) and TSS2 (orange arrowhead). *PP2A* was used as an internal control. **D)** Quantification of longer and shorter *NCP* transcripts in *PIF*-defective mutants. Quantification was performed using 3 independent 5′ RACE-PCR described in **C)**. Blue and orange boxes indicate percentage of quantified band for the longer and shorter *NCP* transcripts, respectively. Error bars indicate Sd of the 3 biological replicates.

We next investigated whether the presence of the *NCP-L* transcript under the WL was influenced by light intensity. We performed 5′ RACE analysis on seedlings grown under 70 *µ*mol m^−2^ s^−1^ red light (R70), 20 *µ*mol m^−2^ s^−1^ red light (R20), or dark conditions, using 3 independent biological replicates. We then quantified the percentages of the 2 *NCP* transcripts. The *NCP-L* transcript, which includes TSS1, was strongly expressed under the R70 conditions, showed reduced expression under R20, and was further reduced in the dark (Fig. 1A). In contrast, the *NCP-S* transcript was detected in the dark and under R20, but it was absent under R70 conditions (Fig. 1A). More than 60% of the transcripts produced in the dark originated from TSS2, while over 70% of the transcripts in R20 and 100% of the transcripts in R70 were generated with TSS1 (Fig. 1B). These results indicate that alternative promoter selection in *NCP* is regulated by red light intensity.

Given that the central mechanism of light signaling involves repressing the levels and activities of a family of antagonizing transcription factors called PIFs, we next asked whether PIFs regulate the light-dependent alternative TSS selection in *NCP.* We analyzed the *NCP* transcript variants in *pif1, pif3*, and *pifq* (*pif1 pif3 pif4 pif5*) mutants grown in the dark. The *NCP-L* transcript level increased marginally in the *pif1* and *pif3* single mutants but increased substantially in the *pifq* mutant (Fig. 1C). In contrast, the *NCP-S* transcript was absent in the *pifq* mutant (Fig. 1C). In Col-0 under dark conditions, the *NCP-S* transcript accounted for ∼60% of total *NCP* transcripts, but this was reduced to ∼25% in both the *pif1* and *pif3* mutants and completely absent in the *pifq* mutant (Fig. 1D). Taken together, these results demonstrate that PHY-mediated red light signaling controls *NCP* transcription initiation in a PIF-dependent manner, favoring TSS1 usage under higher light conditions and TSS2 under lower red light or dark conditions.

### NCP isoforms localize to different subcellular compartments

Although NCP has been previously reported as a dual-targeted protein (Yang et al. 2019), the mechanism underlying its dual targeting remained unclear. The *NCP-L* transcript encodes an isoform containing both a cTP and a predicted NLS (amino acids 118 to 145), whereas the *NCP-S* transcript encodes an isoform containing only the NLS, as the 2nd methionine (start codon) is located at the 56th residue of the NCP-L coding sequence (Supplementary Fig. S3). The predicted cTP region of NCP varies slightly by algorithm, ranging from residues 1 to 48 by ChloroP (Emanuelsson et al. 1999) to 1 to 61 by Localizer (Sperschneider et al. 2017). To determine the minimal N-terminal region required for chloroplast targeting, we fused a series of NCP N termini (N48, N60, N70, and N80) to yellow fluorescent protein (YFP). We assessed their subcellular localization in Arabidopsis Col-0 protoplasts (Supplementary Fig. S4). N48-YFP localized exclusively to the cytoplasm, whereas N60-YFP was detected in both the cytoplasm and chloroplasts. In contrast, both N70-YFP and N80-YFP localized exclusively to chloroplasts. These results indicate that the N-terminal 1 to 70 region is sufficient for exclusive chloroplast localization, whereas the 1 to 60 region is insufficient, suggesting that the cTP cleavage site critical for chloroplast import may reside within residues 60 and 70 (Supplementary Fig. S4).

**Figure 2.**
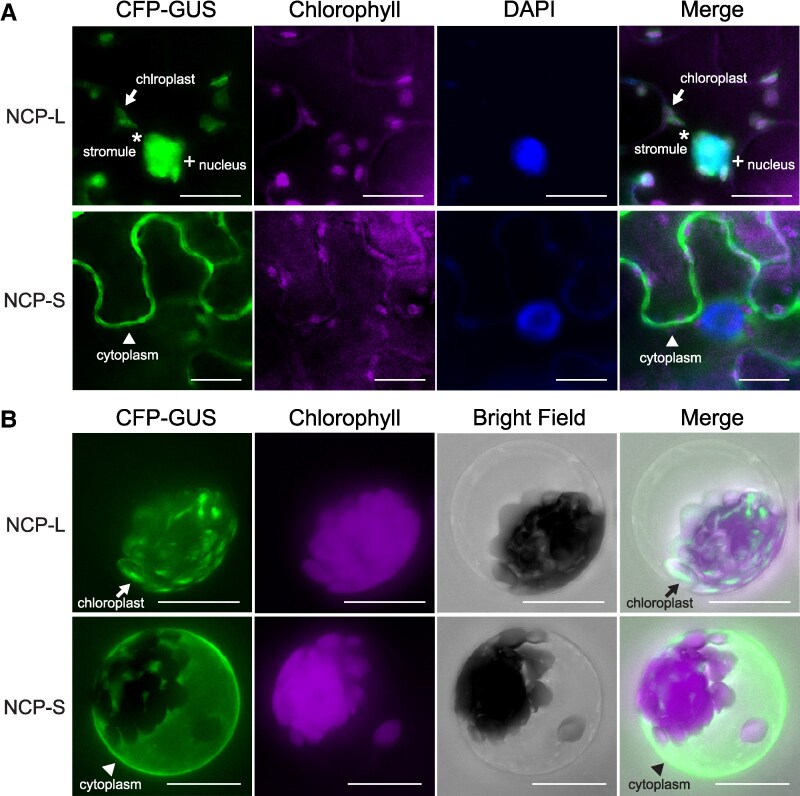
NCP-L and NCP-S protein isoforms show different subcellular localization. **A)** Distinct localization of NCP-L and NCP-S protein isoforms. The *UBQ10pro:NCP-L-CFP-GUS* and *UBQ10pro:NCP-S-CFP-GUS* fusion constructs were expressed transiently in *N. benthamiana* leaves. Fluorescence microscopy images of epidermal cells were visualized. DAPI was used to stain nuclei. Arrows indicate chloroplasts. Asterisks denote stromules. Plus signs (+) indicate nuclear signals. Arrowheads indicate cytoplasmic signals. Chlorophyll, autofluorescence. Scale bars, 20 *μ*m. **B)** Subcellular localization of NCP-L and NCP-S isoforms in Arabidopsis mesophyll protoplasts. The same constructs used in **A)** were expressed transiently in Arabidopsis protoplasts and visualized by fluorescence microscopy. Arrows indicate CFP-GUS signals from chloroplasts. Arrowhead denotes cytoplasmic signals. Scale bars, 20 *μ*m.

To investigate the subcellular localization of NCP-L and NCP-S, we expressed these 2 protein isoforms fused to cyan fluorescent protein (CFP) in *N. benthamiana* leaves. NCP-L-CFP localized predominantly to chloroplasts and also to the nucleus, particularly when the nucleus was surrounded by chloroplasts (Supplementary Figs. S5A and S6A). Interestingly, NCP-L-CFP was observed in stromules (stroma-filled tubular extensions from chloroplasts) that often connected to the nucleus or other chloroplasts (Fig. 2A; Supplementary Fig. S6B). In contrast, NCP-S-CFP localized to the cytoplasm and nucleus, but not to chloroplasts (Supplementary Fig. S5A). Immunoblot analysis showed that the mature NCP-L-CFP was comparable in size with, or slightly smaller than, NCP-S-CFP (Supplementary Fig. S5B). Both mature NCP-L-CFP and NCP-S-CFP appeared as single bands with distinctly higher molecular weights than the chloroplast-targeted CFP alone, cTP-CFP (Supplementary Fig. S5B), confirming the integrity of both fusion proteins in *N. benthamiana* leaves.

Given that the molecular weight of NCP-S-CFP (∼61 kDa) is close to the passive diffusion limit of nuclear pore (around 60 kDa) (Wang and Brattain 2007), we sought to determine whether its nuclear localization was due to passive diffusion. To test this, we fused CFP and GUS to both NCP isoforms, creating fusion proteins large enough to block passive diffusion. The results showed that NCP-S-CFP-GUS localized exclusively to the cytoplasm in 100% of cells (Fig. 2A), with nuclear localization observed in <5% of cells, compared with ∼30% for NCP-S fused with CFP alone (NCP-S-CFP) (Supplementary Fig. S7, A to C). These results suggest that the nuclear localization of NCP-S is primarily due to passive diffusion and that the protein predominantly resides in the cytoplasm. In contrast, NCP-L-CFP-GUS localized to both chloroplasts and the nucleus, and stromule localization was also observed (Fig. 2A). Quantification revealed that both NCP-L-CFP and NCP-L-CFP-GUS primarily localize to the chloroplast, while retaining 80% to 90% of their signal in the nucleus (Supplementary Fig. S6, C and D).

We also examined the subcellular localization of NCP isoforms in Arabidopsis mesophyll protoplasts. NCP-S-CFP-GUS signals were predominantly detected in the cytoplasm (Fig. 2B), consistent with our observation in *N. benthamiana*. NCP-L-CFP-GUS localized exclusively to chloroplasts (Fig. 2B); however, stromules were not observed in these cells, in agreement with previous reports that stromule formation is not typically induced in mesophyll protoplasts without exogenous salicylic acid treatment (Caplan et al. 2015). Taken together, these results indicate that the NCP-S isoform localizes to the cytoplasm, while the NCP-L isoform is targeted to chloroplasts and is often detected in the nucleus, possibly through stromule-mediated translocation.

### The NCP-L isoform rescues both the chloroplast and nuclear phenotypes of the *ncp-10* mutant

**Figure 3.**
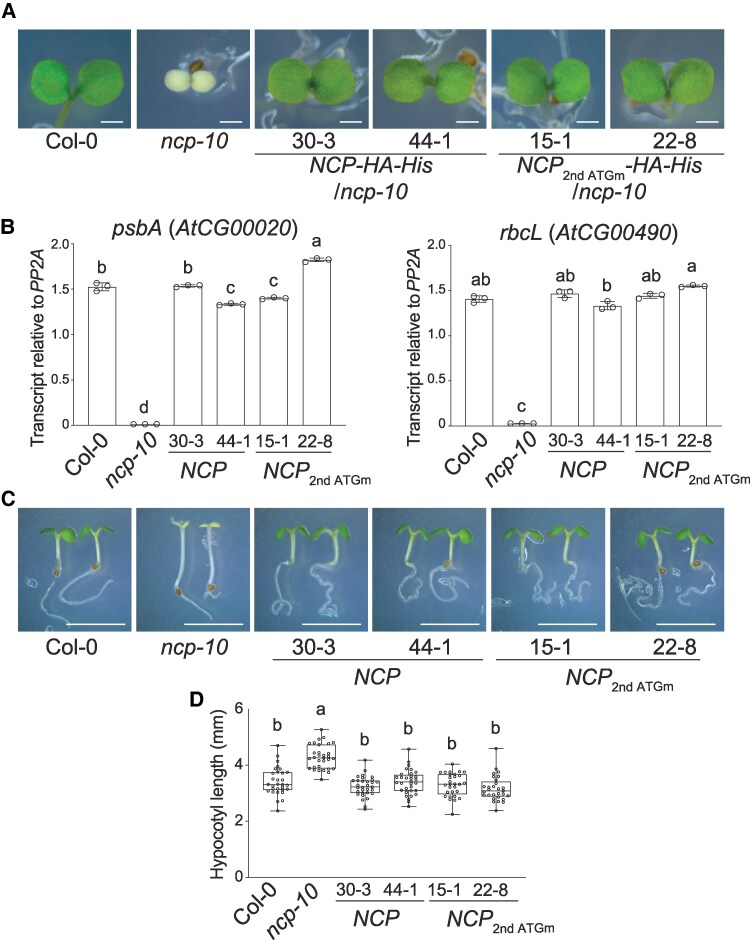
The NCP-L isoform is responsible for chloroplast biogenesis and hypocotyl elongation. **A)** The *ncp-10* albino phenotype is rescued by expressing the wild-type NCP-L isoform (*NCPpro:NCP-HA-His*) and NCP-L with the 2nd ATG mutated NCP 2nd ATGm-HA-His. Col-0, *ncp-10*, *NCPpro:NCP-HA-His/ncp-10*, and *NCPpro:NCP 2nd ATGm-HA-His/ncp-10* seedlings were grown under 20 *μ*mol m^−2^ s^−1^ R light for 4 d. Scale bars, 1 mm. **B)** Transcript levels of PEP-dependent genes in *NCP 2nd ATGm-HA-His ncp-10* plants. Seedlings were grown in 20 *μ*mol m^−2^ s^−1^ R light for 4 d before harvesting whole seedlings for RNA extraction. Transcript levels were examined using RT-qPCR. Different letters denote statistically significant differences in the transcript levels (ANOVA, Tukey's honestly significant difference (HSD), *P* ≤ 0.001). Error bars represent the Sd of 3 biological replicates. **C)** The *ncp-10* tall hypocotyl phenotype is rescued by expressing the wild-type NCP-L isoform (*NCPpro:NCP-HA-His*) and NCP-L with the 2nd ATG mutated *NCP 2nd ATGm-HA-His*. Col-0, *ncp-10*, *NCPpro:NCP-HA-His/ncp-10*, and *NCPpro:NCP 2nd ATGm-HA-His/ncp-10* seedlings were grown under 50 *μ*mol m^−2^s^−1^ R light for 4 d. Scale bars, 5 mm. **D)** Box-and-whisker plots showing hypocotyl measurements of the seedlings in **C)**. Boxes indicate the 25th to 75th percentiles with median values shown as horizontal lines; whisker extends to the minimum and maximum values. No outliers are detected. Sample size (*n*): Col-0 (30), *ncp-10* (31), *NCP-HA-His/ncp-10* #30-3 (31), *NCP-HA-His-ncp-10* #44-1 (32), *NCP 2nd ATGm-HA-His/ncp-10* #22-8 (32), and *NCP 2nd ATGm-HA-His/ncp-10* #15-1 (29). Different letters represent significant differences (*P* ≤ 0.001, 1-way ANOVA with posthoc Tukey's HSD test).

To investigate how the distinct subcellular localization of NCP-L and NCP-S isoforms relates to their biological functions, we generated transgenic plants expressing a ∼4.9-kb *NCP* genomic fragment fused to an hemagglutinin (HA)-histidine (His) tag. To selectively express the NCP-L isoform, we introduced a point mutation at the 2nd ATG codon (ATG to TTG) (Supplementary Fig. S3), thereby preventing translation of the NCP-S isoform (*NCPpro:NCP* 2nd ATGm*-HA-His*/*ncp-10*). As a control, we used transgenic plants expressing the wild-type *NCP* genomic fragment (*NCPpro:NCP-HA-His*/*ncp-10*), which fully rescued the albino phenotype and restored PEP-dependent *psbA* and *rbcL* gene expression in the *ncp-10* (Fig. 3, A and B). Notably, the *NCP* 2nd ATGm transgenic plants also fully rescued both the albino phenotype and the defects in plastid gene expression, indicating that the NCP-L isoform is sufficient to restore PEP activity and chloroplast development.

To evaluate the nuclear function of NCP-L, we examined hypocotyl elongation under red light, as the *ncp-10* mutant exhibits a long hypocotyl phenotype due to impaired phyB signaling (Yang et al. 2019). The tall hypocotyl phenotype of *ncp-10* was also fully rescued in both the wild-type *NCP* and *NCP 2nd ATGm* transgenic plants (Fig. 3, C and D), indicating that nuclear-localized NCP-L is sufficient to restore phyB-mediated signaling. Together, these results underscore the functionality of NCP-L and raise questions about the biological functions of the NCP-S isoform.

### The NCP-S isoform neither rescues the nuclear nor plastidial function of NCP

**Figure 4.**
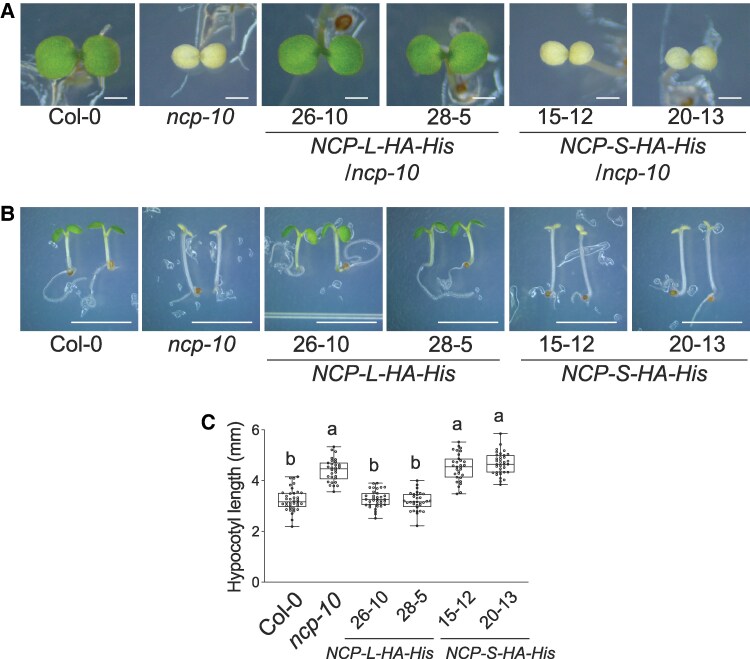
The NCP-S fails to rescue tall-and-albino phenotypes of *ncp-10*. **A)** The NCP-S fails to rescue the *ncp-10* albino phenotype. Col-0, *ncp-10*, *UBQ10pro:NCP-L-HA-His/ncp-10*, and *UBQ10pro:NCP-S-HA-His/ncp-10* seedlings were grown under 20 *μ*mol m^−2^ s^−1^ R light condition for 4 d. Scale bars, 1 mm. **B)** Expression of NCP-S does not complement the tall hypocotyl phenotype of the *ncp-10* mutant. Col-0, *ncp-10*, *UBQ10pro:NCP-L-HA-His/ncp-10*, and *UBQ10pro:NCP-S-HA-His/ncp-10* seedlings were grown under 50 *μ*mol m^−2^s^−1^ R light for 4 d. Scare bars, 5 mm. **C)** Box-and-whisker plots showing hypocotyl measurements of the seedlings in **B)**. Boxes indicate the 25th to 75th percentiles with median values shown as horizontal lines; whisker extends to the minimum and maximum values. No outliers are detected. Sample size (*n*): Col-0 (37), *ncp-10* (30), *NCP-L-HA-His/ncp-10* #26-10 (35), *NCP-L-HA-His/ncp-10* #28-5 (32), *NCP-S-HA-His/ncp-10* #15-12 (31), and *NCP-S-HA-His/ncp-10* #20-13 (35). Different letters represent significant differences (*P* ≤ 0.001, 1-way ANOVA with posthoc Tukey's HSD test).

To investigate the biological function of the NCP-S isoform, we generated transgenic plants expressing the NCP-S coding sequence fused with an HA-His tag under the control of the *UBQ10* promoter in the *ncp-10* mutant background. As a control for comparison, we also generated transgenic plants expressing the NCP-L isoform in *ncp-10*. Consistent with results from *NCP 2nd ATGm-HA-His* lines in Fig. 3, transgenic plants expressing the NCP-L isoform fully rescued the albino and tall hypocotyl phenotypes of *ncp-10* (Fig. 4). In contrast, transgenic plants overexpressing the NCP-S isoform failed to complement either the albino phenotype (Fig. 4A) or the tall hypocotyl phenotype of *ncp-10* (Fig. 4, B and C), indicating that NCP-S is not sufficient to fulfill either the chloroplast or nuclear functions of NCP. Since *NCP-S* transcript levels are known to accumulate under dark conditions, we also tested whether overexpression of *NCP-S* might affect hypocotyl elongation in darkness. However, dark-grown seedlings of *NCP-S* overexpression lines showed hypocotyl lengths comparable with those of Col-0, suggesting that NCP-S does not exert any detectable biological activity under these conditions (Supplementary Fig. S8). Taken together, these findings indicate that the NCP-S isoform is functionally inactive in photomorphogenesis, likely due to cytoplasmic localization.

### The NCP-S isoform is degraded via the 26S proteasome-dependent pathway

**Figure 5.**
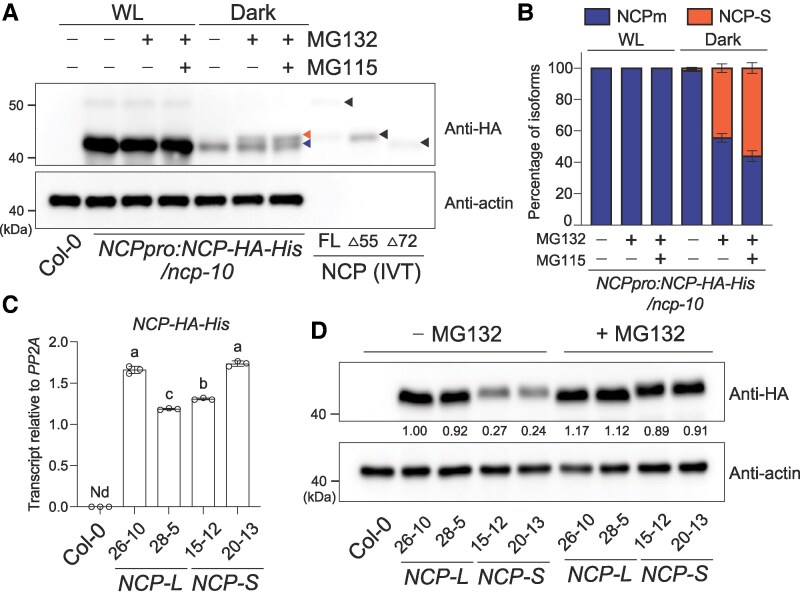
The NCP-S isoform is degraded via the 26S proteasome-dependent pathway. **A)** 26S proteasome-dependent degradation of NCP-S protein. Three-day-old WL or dark-grown *NCPpro:NCP-HA-His/ncp-10* seedlings were incubated without and with 50 *μ*m MG132 and MG112 for 24 h under the same light conditions before extracting total proteins. An anti-HA antibody was used to detect NCP-HA-His protein isoforms. Actin was detected similarly as the loading control. In vitro translated (IVT) NCP full-length (FL) and N-terminal truncation fragments fused with HA-His were used as molecular size controls (black arrowheads). NCP-S-HA-His was marked as an orange arrowhead (left upper). Blue arrowhead (left lower) denotes NCPm-HA-His. **B)** Quantifications from 3 independent blots of *NCPpro:NCP-HA-His/ncp-10* seedling samples shown in **A)** were averaged. Blue and orange boxes indicate the percentage of quantified bands for NCPm-HA-His and NCP-S-HA-His protein isoforms, respectively. Error bars indicate Sd. **C)** Transcript levels of *NCP-HA-His* transgene. Col-0, *UBQ10pro:NCP-L-HA-His/ncp-10*, and *UBQ10pro:NCP-S-HA-His/ncp-10* seedlings were grown under 50 *μ*mol m^−2^ s^−1^ R light condition for 4 d and harvested for total RNA extraction. Transcript levels were examined using RT-qPCR. Different letters denote statistically significant differences in the transcript levels (ANOVA, Tukey's HSD, *P* ≤ 0.001). Error bars represent the Sd of 3 biological replicates. Nd, not detectable. **D)** NCP-S degradation by 26S proteasome. Three-day-old Col-0, *UBQ10pro:NCP-L-HA-His/ncp-10*, and *UBQ10pro:NCP-S-HA-His/ncp-10* seedlings were incubated without and with 50 *μ*m MG132 for 24 h before extracting total proteins from whole seedlings. Anti-HA antibody was used to detect NCPm-HA-His and NCP-S-HA-His proteins. Actin was used as a loading control. Numbers indicate the relative protein level normalized to the actin level.

To investigate the functional deficiency of NCP-S in rescuing the *ncp-10* phenotype, we examined its protein abundance *in planta*. To quantify the levels of NCP-L and NCP-S isoforms under native conditions, we first measured transcript levels specific to either *NCP-L* or to both *NCP-L* and *NCP-S* in the *NCPpro:NCP-HA-His/ncp-10* lines under both WL and dark conditions (Supplementary Fig. S9). We found that the transcript levels were comparable between the transgenic line and wild-type (Col-0) (Supplementary Fig. S9). Subsequently, we performed immunoblotting with anti-HA antibodies on the selected *NCPpro:NCP-HA-His/ncp-10* line. The results showed that the NCP-S protein is not detected under light conditions (Fig. 5A). Despite 5′ RACE confirming transcription of both isoforms in darkness (Fig. 1), NCP-S protein was only weakly detectable in darkness (Fig. 5A). These findings led us to hypothesize that the NCP-S protein is unstable and actively degraded *in planta*.

Given that mislocalized proteins are often targeted by the ubiquitin-proteasome system (UPS) (Ling et al. 2019), we next investigated whether the cytoplasm-localized NCP-S protein was degraded through the 26S proteasome pathway. To test this, we treated the transgenic seedlings with MG132 and MG115, potent 26S proteasome inhibitors. NCP-S protein levels were substantially restored in dark-grown seedlings upon inhibitor treatments. In contrast, the mature form of NCP (NCPm) derived from NCP-L levels remained largely unaffected (Fig. 5, A and B). We found that NCP-S migrates similarly to NCPΔ55 (Fig. 5A; Supplementary Fig. S3), while the size of NCPm is equivalent to NCPΔ72 (Fig. 5A). The chloroplast transit peptide of NCP is mapped to the region between amino acids 60 and 70 (Supplementary Fig. S4), indicating only a minimal expected size difference between NCPm and NCP-S. However, the distinct migration shift observed in Fig. 5A suggests that additional proteolytic processing may affect NCPm mobility, as has also been proposed for chloroplast-imported proteins (Nevarez et al. 2017).

To further validate this degradation, we examined protein levels in *NCP-L* and *NCP-S* overexpression lines. In these lines, the transcript levels of the *NCP-L-HA-His* and *NCP-S-HA-His* transgenes were similarly overexpressed by the *UBQ10* promoters (Fig. 5C). Consistent with our previous observations, NCP-S protein levels were markedly lower than those of the mature form of NCP-L (NCPm) in the absence of MG132 treatment (Fig. 5D). Remarkably, MG132 treatment selectively increased NCP-S protein accumulation. However, NCPm protein level remained largely unchanged (Fig. 5D). Together, these results indicate that the mislocalized NCP-S protein in the cytoplasm is degraded via the 26S proteasome-dependent pathway.

### Chloroplast-localized NCPm is required for nucleoid localization of PEP core proteins

The PEP complex is essential for chloroplast biogenesis and is known to localize to plastid nucleoids, which appear as irregularly shaped condensed structures that serve as sites of plastid gene transcription (Powikrowska et al. 2014). Since chloroplast-targeted NCP-L is sufficient to complement the albino phenotype of *ncp-10*, we investigated whether and how the chloroplast-localized NCPm affects the nucleoid localization of PEP complex components.

Since PEP core components such as rpoA and rpoB are encoded by the plastidial genome, they lack an N-terminal cTP. We generated fusion constructs containing the cTP from RBCS1A (Shen et al. 2017) and YFP at the N terminus of either rpoA or rpoB. These constructs (cTP-YFP-rpoA and cTP-YFP-rpoB) were expressed in Arabidopsis protoplasts isolated from wild-type and *ncp-10* albino seedlings (Supplementary Fig. S10A). As a control, we expressed cTP-YFP alone, which localized to plastids in wild-type protoplasts (Supplementary Fig. S10B). The cTP-YFP also localized to plastids in *ncp-10* protoplasts, confirming that plastid protein import remains functional in *ncp-10,* despite reduced chlorophyll autofluorescence (Supplementary Fig. S10B).

**Figure 6.**
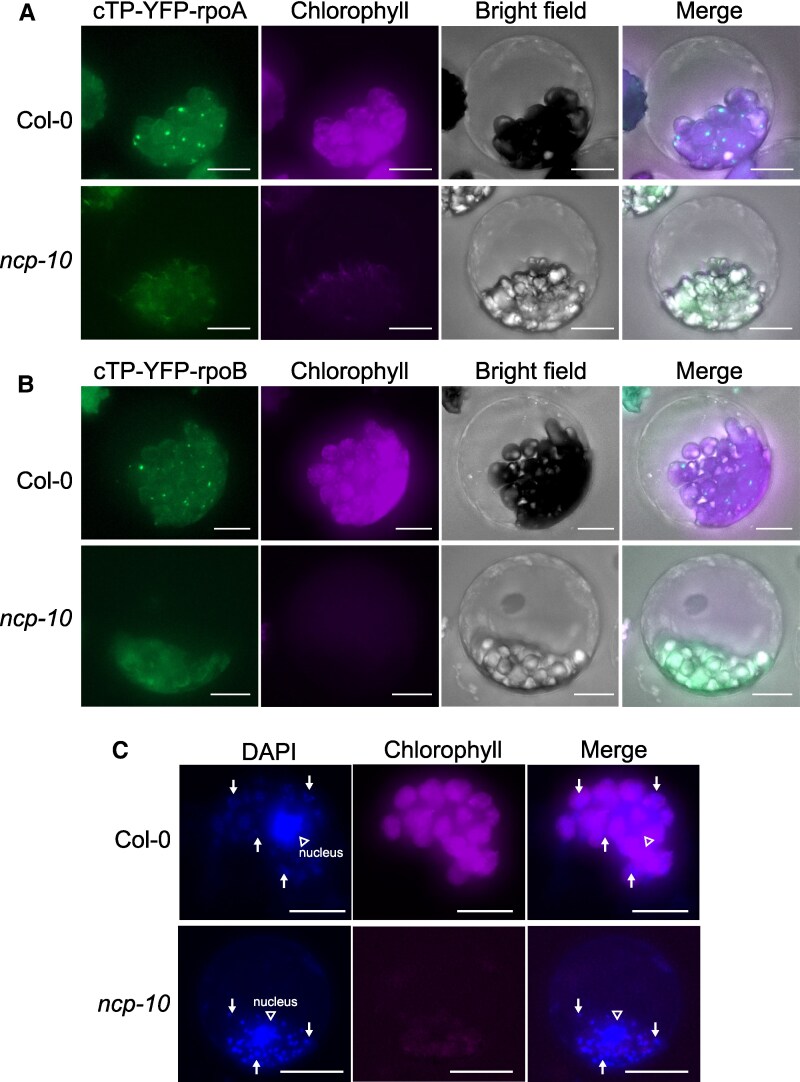
Condensed localization of rpoA and rpoB in nucleoids is impaired in *ncp-10*. **A** and **B)** Impaired localization pattern of rpoA and rpoB in *ncp-10* protoplast. The coding sequence of N-terminal chloroplast transit peptide (cTP) of RBCS1A was transcriptionally fused to 5′ end of YFP-rpoA and YFP-rpoB coding sequences under the control of CaMV 35S promoter. The cTP-YFP-rpoA **A)** and cTP-YFP-rpoB **B)** constructs were expressed transiently in Arabidopsis protoplasts, and the chloroplasts were visualized by fluorescence microscopy. Scale bars, 10 *μ*m. **C)** Nucleoids were visualized by DAPI in Col-0 and *ncp-10* protoplasts. Arrows indicate nucleoids. Empty arrowhead indicates the nucleus. Chlorophyll, autofluorescence. Scale bars, 20 *μ*m.

In wild-type protoplasts, cTP-YFP-rpoA localized to several discrete foci within the chloroplasts (Fig. 6A), consistent with previously reported nucleoid patterns (Powikrowska et al. 2014). To validate that these foci correspond to nucleoids, we performed high-concentration DAPI staining in protoplasts. The cTP-YFP-rpoA signals colocalized with DAPI-stained nucleoids in protoplasts (Supplementary Fig. S11), confirming that the PEP complex localizes to nucleoids. In contrast, cTP-YFP-rpoA exhibited a diffuse distribution throughout the plastids in *ncp-10* protoplasts (Fig. 6A). Similarly, cTP-YFP-rpoB showed punctate localization in wild-type chloroplasts but appeared a dispersed pattern in *ncp-10* plastids (Fig. 6B), indicating that nucleoid localization of PEP components is disrupted in the absence of functional NCPm.

To determine whether this dispersed localization was due to altered nucleoid organization in *ncp-10*, we stained plastids with DAPI. Nucleoids were still present in *ncp-10* protoplasts but appeared slightly enlarged relative to wild-type (Fig. 6C), consistent with previous observations in the *ncp-10/svr4-like* mutant (Powikrowska et al. 2014). The DAPI-stained *ncp-10* nucleoids remained condensed, in contrast to the diffuse localization pattern observed for rpoA and rpoB. Since the PEP complex fails to assemble in *ncp-10* (Yang et al. 2019), these results suggest that chloroplast-localized NCPm is required not only for the PEP assembly but also for proper nucleoid association of PEP core components.

### NCP's nuclear function requires its prior chloroplast localization

Our data showed that chloroplast-targeted NCP-L can complement both the albino and tall hypocotyl phenotypes of *ncp-10*. Furthermore, NCP-L accumulation in the nucleus was observed specifically in cells where the nucleus is surrounded by chloroplasts, and stromules were present in these chloroplasts. These observations led us to hypothesize that NCP's localization to the nucleus depends on its prior targeting to chloroplasts.

**Figure 7.**
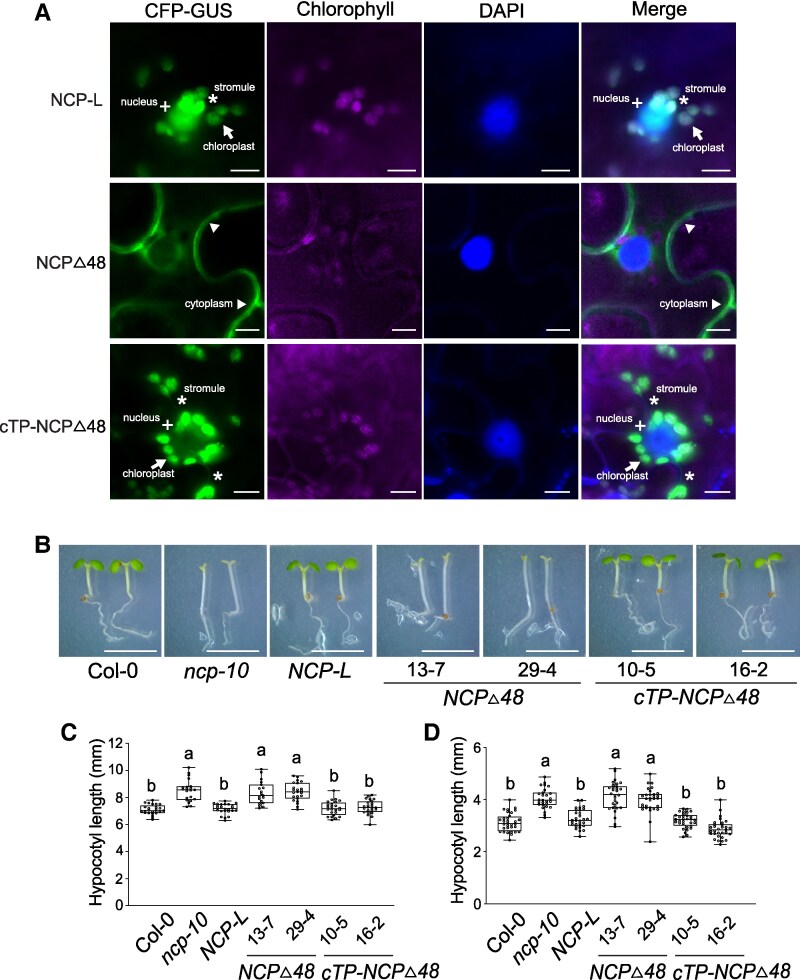
NCP requires its prior chloroplast localization to rescue the tall hypocotyl phenotype of *ncp-10*. **A)** Defect of nuclear accumulation of NCPΔ48 is restored by fusing the chloroplast transit peptide (cTP) of RBCS1A. The *UBQ10pro:NCP-L-CFP-GUS*, *UBQ10pro:NCPΔ48-CFP-GUS*, and *UBQ10pro:cTP-NCPΔ48-CFP-GUS* fusion constructs were expressed transiently in *N. benthamiana* leaves. The nucleus and chloroplasts were visualized by fluorescence microscopy. Arrows indicate chloroplasts. Asterisks and plus signs (+) denote stromules and the nucleus, respectively. Arrowheads indicate signals from the cytoplasm. Chlorophyll, autofluorescence. Scale bars, 10 *μ*m. **B)** Fusing the chloroplast transit peptide (cTP) of RBCS1A with NCPΔ48 rescues the tall hypocotyl phenotype of the *ncp-10*. Col-0, *ncp-10*, *NCP-L-HA-His/ncp-10* #28-5, *UBQ10pro:NCPΔ48-HA-His/ncp-10*, and *UBQ10pro:cTP-NCPΔ48-HA-His/ncp-10* seedlings were grown under 50 *μ*mol m^−2^s^−1^ R light for 4 d. Scare bars, 5 mm. Box-and-whisker plots showing hypocotyl measurements of the seedlings grown under 1 *μ*mol m^−2^s^−1^ R light **C)** and 50 *μ*mol m^−2^s^−1^ R light **D)**. Boxes indicate the 25th to 75th percentiles with median values shown as horizontal lines; whisker extends to the minimum and maximum values. No outliers are detected. Sample size for **C** and **D)** (*n*): Col-0 (25, 32), *ncp-10* (21, 27), *NCP-L-HA-His/ncp-10* #28-5 (24, 30), *NCPΔ48-HA-His/ncp-10* #13-7 (19, 30), *NCPΔ48-HA-His/ncp-10* #29-4 (23, 30), *cTP-NCPΔ48-HA-His/ncp-10* #10-5 (24, 32), and *cTP-NCPΔ48-HA-His/ncp-10* #16-2 (24, 31). Different letters represent significant differences (*P* ≤ 0.001, 1-way ANOVA with posthoc Tukey's HSD test).

To test this, we first analyzed the localization pattern of NCPΔ48, a truncated version of NCP lacking the N-terminal 1 to 48 amino acids required for chloroplast targeting (Supplementary Fig. S4). The NCPΔ48 coding sequence was fused to CFP and GUS, and the resulting NCPΔ48-CFP-GUS construct was transiently expressed in *N. benthamiana* leaves. The NCPΔ48-CFP-GUS signal was detected exclusively in the cytoplasm and was absent from both the chloroplasts and nucleus (Fig. 7A). However, when the cTP of RBCS1A was fused to NCPΔ48, the fusion protein was efficiently targeted to chloroplasts and also detected in the nucleus (Fig. 7A). Interestingly, cTP-NCPΔ48-CFP-GUS was accumulated in stromules extending toward the nucleus, a localization pattern similar to that of NCP-L-CFP-GUS (Fig. 7A). These findings indicate that prior chloroplast localization is required for NCP's nuclear accumulation, potentially through stromule-mediated retrotranslocation.

To further investigate the functional significance of chloroplast localization for nuclear activity, we generated Arabidopsis transgenic lines expressing NCPΔ48-HA-His or cTP-NCPΔ48-HA-His in the *ncp-10* background. NCPΔ48-HA-His failed to rescue either the albino phenotype (Fig. 7B) or the tall hypocotyl phenotype of *ncp-10* both under 1 *µ*mol m^−2^ s^−1^ red light (R1) and 50 *µ*mol m^−2^ s^−1^ red light (R50) conditions (Fig. 7, C and D). In contrast, cTP-NCPΔ48-HA-His fully complemented both phenotypes, restoring chloroplast development and normal hypocotyl growth. We next asked whether overexpression of *NCP-L* was sufficient to enhance phyB signaling. To test this, we analyzed hypocotyl elongation responses in transgenic plants overexpressing *NCP* (*NCPpro:NCP-HA-His*/Col-0, *NCPpro:NCP 2nd ATGm-HA-His*/Col-0, and *UBQ10pro:NCP-L-HA-His*/Col-0). We found that none of these lines showed any significant difference in hypocotyl length under red light across a range of fluence rates (Supplementary Fig. S12, A and B). These findings indicate that while cTP-NCPΔ48-HA-His is sufficient to rescue the nuclear phenotypes in *ncp-10*, its overexpression alone is not sufficient to enhance phyB signaling. Together, these results indicate that its prior chloroplast localization is critical for NCP's nuclear function, supporting a model in which NCP participates in chloroplast-to-nucleus signaling.

### Chloroplast-targeted NCPm translocates to the nucleus possibly through stromules

Stromules are chloroplast-derived dynamic tubules that extend toward the nucleus and enable the transfer of signaling molecules (Hanson and Hines 2018; Mullineaux et al. 2020). Our localization data in *N. benthamiana* showed that the chloroplast-targeted NCPm was frequently detected in stromules, which often extended toward and connected with the nucleus. Therefore, we hypothesized that NCP retrotranslocates to the nucleus to maintain PHY-mediated signaling, potentially via the stromules.

**Figure 8.**
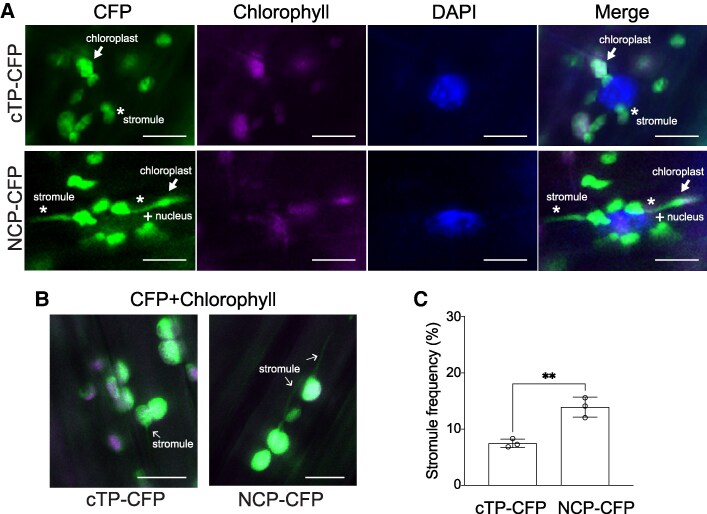
NCP sends the signal back to the nucleus potentially via stromules in Arabidopsis hypocotyl. **A)** NCP-mediated stromule induction in Arabidopsis hypocotyl. The *UBQ10pro:cTP-CFP*/Col-0 and *UBQ10pro:NCP-CFP*/Col-0 plants were grown for 4 d under 50 *μ*mol m^−2^ s^−1^ red light (R50). Fluorescence microscopy images of hypocotyl cells were visualized. DAPI was used for staining nuclei. Arrows and asterisks indicate chloroplasts and stromules, respectively. Plus signs (+) denote signals from the nucleus. Chlorophyll, autofluorescence. Scale bars, 10 *μ*m. **B)** Expressing NCP-CFP exhibits enhanced stromule induction. Seedling growth conditions are as described in **A)**. Fluorescence microscopy images of hypocotyl cells were visualized. Arrows indicate stromules. Scale bars, 10 *μ*m. **C)** Quantification of stromules from experiments described in **A)** and **B)** reveals an enhanced frequency of stromules in NCP-CFP-expressing plants compared with the cTP-CFP control plants. Three biological replicates, each consisting of 11 seedlings, were averaged. Bars indicate Sd. **Indicates a statistically significant difference (Student's *t*-test, *P* < 0.01).

To support the retrotranslocation mechanism, we generated Arabidopsis transgenic lines expressing either RBCS1A cTP fused CFP (cTP-CFP) or NCP-CFP under the control of *UBQ10* promoters in the Col-0 background. In hypocotyl cells expressing cTP-CFP, stromules were rarely detected and appeared short in length, with a frequency below 10% (Fig. 8, A to C). In contrast, *NCP-CFP-*expressing lines displayed more than a twofold increase in stromule frequency (Fig. 8C), including longer stromules frequently extending toward the nucleus (Fig. 8, A and B). These long stromules were often associated with nuclear-localized NCP-CFP signal, supporting a model in which stromule formation facilitates NCP retrotranslocation to the nucleus. Together, our results indicate that NCP initially localizes to chloroplasts and that stromules promote its nuclear accumulation, establishing a retrograde signaling route during early seedling photomorphogenesis.

## Discussion

Interorganellar communication between the nucleus and chloroplasts is essential for establishing and maintaining photomorphogenesis, a developmental program characterized by nuclear regulation of hypocotyl growth and the light-triggered conversion of proplastids or etioplasts into functional chloroplasts (Griffin and Toledo-Ortiz 2022). In this study, we uncover a light-regulated, dual-targeting mechanism of NCP that integrates bidirectional signaling between the nucleus and chloroplasts to coordinate photomorphogenesis in Arabidopsis. Our data show that light regulates alternative transcription initiation in *NCP*, favoring TSS1 via PIF-mediated PHY signaling, which produces the longer NCP-L isoform. NCP-L is targeted to chloroplasts via its N-terminal cTP, where it is processed into the NCPm. Within the plastid, NCPm promotes assembly and nucleoid localization of the PEP complex, initiating transcription of *PhAPGs* and driving chloroplast biogenesis. We further reveal that NCP's nuclear accumulation requires its prior localization to chloroplasts. Once localized to chloroplasts, NCPm retrotranslocates to the nucleus, potentially via stromules, where it potentiates PHY signaling by facilitating the formation of phyB photobodies (Yang et al. 2019), thereby repressing hypocotyl elongation in the light. This chloroplast-to-nucleus retrograde signaling establishes a feedback mechanism that sustains photomorphogenesis. In contrast, under dark conditions, inactive PHYs lead to PIF accumulation, which inhibits TSS1 and shifts *NCP* transcription to TSS2, producing the shorter NCP-S isoform. Lacking the cTP, NCP-S cannot enter plastids and instead accumulates in the cytoplasm, where it is degraded via the 26S proteasome pathway. Although it retains a predicted NLS, NCP-S is predominantly localized in the cytoplasm, suggesting that the predicted NLS may be nonfunctional.

**Figure 9.**
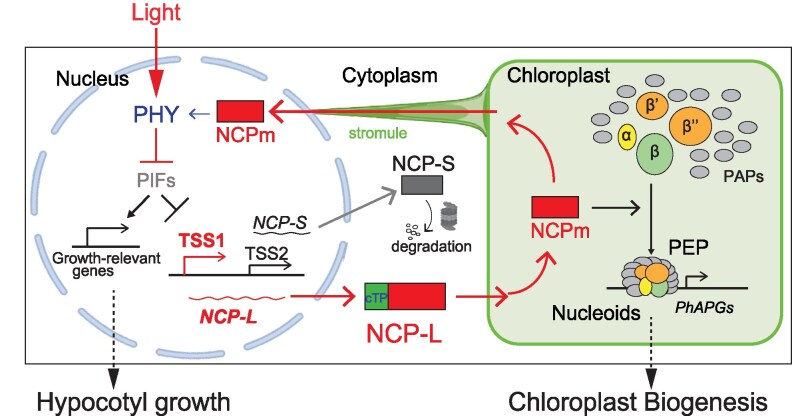
Nucleus–chloroplast interorganellar communication via an NCP-mediated signaling cascade. Light triggers chloroplast differentiation by controlling PIF-dependent alternative promoter usage in *NCP*, producing chloroplast-targeted NCP-L. Upon cleavage of transit peptide and additional proteolytic processing, the NCPm promotes PEP assembly on nucleoids to initiate chloroplast biogenesis. NCPm translocates to the nucleus, potentially through direct connections via stromules. We propose that NCPm sends the signal back to the nucleus to maintain PHY signaling, thereby inhibiting hypocotyl growth during photomorphogenesis.

Together, our findings establish NCP as a light-regulated, dual-localized protein that mediates interorganellar communication in both directions: (i) Anterograde signaling: nuclear control of chloroplast biogenesis via PHY/PIF-regulated alternative promoter selection and plastid-localized NCPm function and (ii) Retrograde signaling: chloroplast to nucleus retrotranslocation of NCPm via stromules, maintaining PHY signaling to continuously inhibit hypocotyl elongation. This dual-targeting, bidirectional signaling model highlights an interorganellar communication mechanism that coordinates gene expression between the nucleus and chloroplasts to establish and sustain photomorphogenesis (Fig. 9). In support of our model, both *NCP-L* transcript levels (Supplementary Fig. S9) and NCPm protein abundance (Fig. 5A) increase significantly under light conditions compared with darkness. This coordinated induction suggests that light promotes NCPm accumulation in chloroplasts as a prerequisite for its retrotranslocation to the nucleus, potentially enabling its nuclear function in photomorphogenesis. To further test this model, future investigations using high-temporal-resolution cell biological approaches are needed to assess the spatiotemporal dynamics of NCPm localization during the dark-to-light transition.

Alternative promoter selection plays a crucial role in generating diverse transcripts that encode proteins with varied functions, subcellular localizations, and transcriptional/translational regulatory mechanisms (Ayoubi and Van De Ven 1996; Huin et al. 2017; Alfonso-Gonzalez and Hilgers 2024). In plants, PHYs have been known to control alternative promoter selection to modulate protein localization as part of the adaptation to the light environment (Ushijima et al. 2017). For example, under light conditions, glycerate kinase (GLYK) localizes to chloroplasts and functions in photorespiration. However, under shade conditions, inactivated PHYs lead to alternative transcription initiation, producing a cytoplasmic GLYK isoform, lacking its cTP, for a cytoplasmic photorespiratory bypass (Ushijima et al. 2017). In line with this study, our data reveal that light-dependent alternative promoter usage causes the *NCP* gene to produce NCP-L, which contains a cTP, under high light conditions, while the NCP-S isoform without a cTP is produced under low light/dark conditions. However, the functional consequences of this mechanism on NCP during photomorphogenesis are different. The mistargeted NCP-S protein, which lacks a cTP due to alternative promoter usage in low light or dark conditions, is degraded by the 26S proteasome-dependent pathway in the cytoplasm. In eukaryotic cells, organelle-targeted proteins are tightly regulated by the protein import pathway and subjected to degradation via the UPS if they are mislocalized or misfolded (Hegde and Zavodszky 2019; Ling et al. 2019). Thus, we propose a molecular mechanism in which alternative promoter usage is linked to the quality control of protein localization. This mechanism ensures that proteins are correctly targeted to their functional locations, while mislocalized proteins are rapidly degraded to prevent dysfunction. From another perspective, alternative promoter usage in *NCP* represents a finely tuned regulatory mechanism in nucleus-to-plastid signaling, controlling the ratio of *NCP-L* and *NCP-S* transcripts to adjust the amount of functional NCP-L protein under fluctuating environmental light conditions. Although NCP-S does not appear to play an essential role in photomorphogenesis, it remains possible that NCP-S could accumulate under certain conditions due to the regulation of alternative promoter usage and degradation pathways. Further investigations are needed to explore the potential roles of NCP-S in the cytoplasm when it does accumulate and whether it may have unknown functions.

Mechanisms for alternative transcription initiation have been proposed at different levels (Ayoubi and Van De Ven 1996; Alfonso-Gonzalez and Hilgers 2024). First, transcription factor binding to specific enhancers in different regions can activate alternative transcription (Wang et al. 2022). Second, structural changes in chromatin via histone modifications or DNA methylation can alter the local architecture and facilitate open chromatin for transcription initiation (Maqbool et al. 2020; Nepal and Andersen 2023). However, the precise mechanism by which PHYs regulate alternative promoter selection remains elusive. We found that light-dependent alternative transcription initiation in *NCP* is dependent on PIFs, as demonstrated in the *pif1*, *pif3*, and *pifq* mutant backgrounds. Notably, a potential PIF-binding E-box (CANNTG) exists in exon 2, close to TSS2, suggesting that PIF proteins might directly bind to this region and prevent transcription initiation from TSS1, while promoting initiation from TSS2. This dual action could explain the shift in TSSs under different light conditions. However, our attempts to verify direct PIF binding to the *NCP* gene via chromatin immunoprecipitation–sequencing were inconclusive when analyzing datasets from PIF1, PIF3, PIF4, or PIF5 (Oh et al. 2009; Hornitschek et al. 2012; Zhang et al. 2013; Pfeiffer et al. 2014). These results suggest that the PIF-dependent regulation of alternative transcription initiation in *NCP* may involve additional transcription factors or chromatin remodelers that work in concert with PIFs.

Functional complementation experiments using a truncated version of NCP lacking the N-terminal 48 residues (NCPΔ48) failed to rescue either the albino or hypocotyl phenotypes of *ncp-10*, whereas a version with the RBCS1A cTP (cTP-NCPΔ48) fully restored both phenotypes. These findings confirm that prior chloroplast localization is required for NCP's nuclear function and suggest that NCPm retrotranslocates to the nucleus to modulate PHY signaling for hypocotyl growth regulation. It is noteworthy that overexpression of *NCP-L* lines did not show any discernible hypocotyl inhibition phenotype, suggesting that NCP is necessary but not rate-limiting for plastid-derived nuclear signaling. This implies that the NCP function is tightly coordinated within broader signaling networks, potentially requiring additional cofactors or reflecting that the level of retrotranslocated nuclear NCPm is limiting under normal conditions.

Stromules are long, thin, tubular extensions from plastids and have been proposed as pathways for the retrotranslocation of molecules to the nucleus (Kwok and Hanson 2004; Caplan et al. 2015; Hanson and Conklin 2020). Our data strongly support the hypothesis that stromules mediate retrograde protein movement from chloroplasts to the nucleus. In Arabidopsis hypocotyls, transgenic lines expressing NCP-CFP showed significantly higher stromule frequency and longer stromules in length compared with control lines expressing cTP-CFP (Fig. 8). These results provide in vivo evidence that NCPm-associated stromules may facilitate retrotranslocation to the nucleus during photomorphogenesis. Although the mechanism by which NCP induces stromules is unclear, reactive oxygen species (ROS) generated by the chloroplast electron transport chain are known to promote stromule formation (Brunkard et al. 2015; Caplan et al. 2015; Foyer and Hanke 2022; Li and Kim 2022). Since chloroplast biogenesis during photomorphogenesis likely elevates ROS production (Fichman et al. 2023), it is possible that the NCP-mediated retrotranslocation via stromules may be coordinated with developmental ROS signaling.

The notion that proteins can move from plastids to the nucleus represents an emerging paradigm in plant signaling during photomorphogenesis. Similar to NCP, other dual-targeted proteins such as PAP5/HMR, PAP8/pTAC6, RCB, and Whirly1 (WHY1) have been implicated in regulating both chloroplast and nuclear functions (Grabowski et al. 2008; Nevarez et al. 2017; Yoo et al. 2019; Chambon et al. 2022). Indeed, the dual-targeted proteins are critical for chloroplast biogenesis and also modulate PHY signaling by promoting the formation of phyB photobodies in the nucleus (Galvão et al. 2012; Yang et al. 2019; Yoo et al. 2019; Liebers et al. 2020; Willige et al. 2024). However, the biological meaning of chloroplast-to-nucleus retrotranslocation and PHY signaling still remains an intriguing question. We propose that during the dark-to-light transition, chloroplasts transmit a signal to the nucleus possibly through stromules, promoting PHY signaling through the stabilization of large phyB photobodies and coordinating chloroplast and nuclear responses during photomorphogenesis. Future studies are poised to shed light on the underlying molecular mechanisms and their broader biological implications.

## Materials and methods

### Plant materials and growth conditions

The *ncp-10* (Col-0 background) mutant has been described previously (Yang et al. 2019). The Arabidopsis (*A. thaliana*) mutants *pif1-2* (Huq et al. 2004), *pif3-1* (Kim et al. 2003), and *pifq* (Leivar et al. 2008), all in the Col-0 background, were used for the 5′ RACE experiment. Additional Arabidopsis transgenic lines generated in this study are described below. Seeds were surface-sterilized and plated on half-strength ½ MS media with Gamborg's vitamins (MSP06, Caisson Laboratories, North Logan, UT, USA), 0.5 mm MES pH 5.7, and 0.8% agar (w/v) (A037, Caisson Laboratories). Seeds were stratified in the dark at 4 °C for 4 d. Seedlings were grown at 21 °C in an LED chamber (Percival Scientific) under the indicated light conditions. For true-dark treatment, stratified seeds were preilluminated with far-red light (10 *μ*mol·m^−2^·s^−1^) for 3 h before transferring to darkness. Fluence rates of light were measured using a Li-180 Spectrometer (LI-COR Biosciences).

### Plasmid construction and generation of transgenic plants

To generate the *NCPpro*:*NCP*-*HA*-*His* fusion construct, 3× HA and 6× His coding sequences were fused in-frame to the 3′ end of the *NCP* genomic sequence driven by its own promoter, consisting of a ∼4.9-kb sequences. The fusion construct was subcloned into the EcoRI and BamHI sites of the *pJHA212* vector (Yoo et al. 2005) using HiFi DNA assembly (New England Biolabs, Ipswich, MA, USA). The second ATG of the *NCP* coding sequence in the *pJHA212*-*NCPpro*:*NCP*-*HA*-*His* was mutated to TTG to block the translation of the NCP-S isoform, resulting in *pJHA212*-*NCPpro:NCP*_2nd ATGm_-*HA*-*His*. Full-size NCP long (L) and short (S) coding sequences were fused in-frame to the 5′ end of an *HA-His*-coding sequence, and the fusion protein was overexpressed using the *UBQ10* promoter, and ligated into the BamHI and PstI sites of the *pJHA212* vector to generate the *UBQ10pro:NCP*-*L*-*HA*-*His* and *UBQ10pro:NCP*-*S*-*HA*-*His* transgenic plants. Transgenic lines expressing the indicated *NCP*-*HA*-*His* fusion constructs were generated by transforming *ncp-10* plants with *Agrobacterium tumefaciens* strain GV3101 harboring the constructs (Clough and Bent 1998). Multiple independent lines from the T1 generation were selected on MS medium containing 100 *μ*g/mL gentamycin and 2% sucrose. Plants with *ncp-10* mutations were selected in the T1 generation. T2 lines with a single-locus transgene insertion were selected based on a 3:1 segregation ratio for gentamycin resistance. T3 generation plants homozygous for the transgene were used for further experiments. For the *UBQ10pro:NCP*-*S*-*HA*-*His* transgenic lines, plants homozygous for the transgene and heterozygous for the *ncp-10* mutation in the T3 generation were used for subsequent experiments. To generate *UBQ10pro:cTP-NCPΔ48-HA-His* plants, the N-terminal chloroplast transit peptide sequence of RBCS1A was fused in-frame to the 5′ end of the NCPΔ48-HA-His construct. The resulting fusion was then subcloned into the BamHI and PstI sites of the *pJHA212* vector under the control of the *UBQ10* promoter. CFP coding sequences were fused in-frame to the 3′ ends of the *RBCS1A cTP* and *NCP* gene, respectively, and the fusions were ligated into the BamHI and PstI sites of the *pJHA212* vector under the control of the *UBQ10* promoters, resulting in *UBQ10pro:cTP-CFP*/Col-0 and *UBQ10pro:NCP-CFP*/Col-0 plants. Primers used for plasmid construction are listed in Supplementary Table S1. Primers used for genotyping assays are listed in Supplementary Table S2.

The *UBQ10pro:NCP*-*L(S)*-*CFP* and *UBQ10pro:NCP*-*L(S)*-*CFP*-*GUS* fusion constructs used for the transient expression assay in *N. benthamiana* and Arabidopsis protoplasts were prepared by amplifying the NCP-L and NCP-S coding sequences. Each fragment was fused in-frame to the 5′ end of either CFP or CFP-GUS coding sequences. The fusion constructs were ligated into the BamHI and PstI sites of the *pJHA212* vector under the control of the *UBQ10* promoters. To generate the *UBQ10pro:NCPm-CFP-GUS* construct, the NCPm-coding sequence was fused in-frame to the 5′ end of CFP- and GUS-coding sequences, and the fusion was inserted into the BamHI and PstI sites of the binary *pJHA212* vector under the *UBQ10* promoter. The coding sequences of the N-terminal chloroplast transit peptide (cTP) of RBCS1A were transcriptionally fused to the 5′ end of NCPm-CFP-GUS-coding sequences under the same *UBQ10* promoter, resulting in *UBQ10pro:cTP-NCPm-CFP-GUS*.

A series of deletion constructs was generated to examine the N-terminal chloroplast shuttling of NCP in Arabidopsis protoplasts. The N48-YFP, N60-YFP, N70-YFP, and N80-YFP fusions were ligated into the XbaI and KpnI sites of the *p326-HAN* vector (Jin et al. 2001) under the CaMV 35S promoter. For preparing cTP-YFP-rpoA and cTP-YFP-rpoB constructs, the coding sequence of N-terminal chloroplast transit peptide (cTP) of RBCS1A was transcriptionally fused to 5′ end of YFP-rpoA and YFP-rpoB coding sequences and ligated into the XbaI and KpnI sites of the *p326-HAN* vector under the control of CaMV 35S promoter.

For generating in vitro translated full-length NCP and N-terminally truncated NCP fragments, a series of NCP-coding sequences were fused in-frame to the 5′ end of an *HA-His*-coding sequence and ligated into the EcoRI and BamHI sites of the *pCMX-PL2* vector.

### 5′ RACE analysis

One microgram of total RNA was used for first-strand cDNA synthesis for 5′ rapid amplification of cDNA ends (5′ RACE) analysis, which was performed using a SMARTer RACE 5′/3′ Kit (Clontech Laboratories) according to the manufacturer's instructions. The obtained cDNA was subjected to 5′ RACE-PCR using SeqAmp DNA Polymerase (Takara Bio USA). Primers used for 5′ RACE-PCR and the number of PCR cycles are shown in Supplementary Table S3. The 5′ RACE-PCR products were separated by electrophoresis on a 1% agarose gel, and the DNA contained therein was excised and extracted using a Zymoclean Gel DNA Recovery Kit (Zymo Research). Each transcript extracted was then subcloned into a pRACE cloning vector (Clontech Laboratories) with an In-Fusion Cloning Kit (Clontech Laboratories). Plasmid DNA was purified using a ZR Plasmid Miniprep-Classic Kit (Zymo Research) and sequenced by the University of Utah DNA sequencing core center. At least 3 independent 5′ RACE clones from biological triplicate samples were sequenced to determine the transcription initiation sites.

### Transient expression in Arabidopsis protoplasts

Mesophyll protoplasts were isolated from mature Arabidopsis leaves according to the procedure (Yoo et al. 2007) to define the subcellular localization of NCP-L and NCP-S protein isoforms. To isolate protoplasts from seedlings for nucleoid localization of the core component proteins, Col-0 and *ncp-10* plants were grown on ½ MS plates supplemented with 3% sucrose for 3 wk under long day conditions (16 h light/8 h dark cycles), and the whole seedlings were preincubated in 0.4 m mannitol solution for 1 h. Overnight incubation of the seedlings was performed in the enzyme solution (20 mm MES pH 5.7, 1.5% [w/v] cellulase R10, 0.4% [w/v] macerozyme R10, 20 mm KCl, 10 mm CaCl_2_, and 0.1% bovine serum albumin). High-purity plasmid DNA was prepared using PureLink HiPure Plasmid Midiprep Kit (Thermo Fisher Scientific). Arabidopsis mesophyll protoplast transfection was performed by a PEG–calcium transfection method (Yoo et al. 2007). After transfection, to visualize the localization of NCP-L and NCP-S protein isoforms, the transfection reactions were incubated in WL (80 *μ*mol m^−2^ s^−1^) at room temperature for 24 h. For visualizing cTP-YFP-rpoA and cTP-YFP-rpoB expression in chloroplast nucleoids, the transfection reactions were incubated at room temperature for 48 h under the same light conditions. For DAPI staining, 10 *μ*L of 10 *μ*g/mL DAPI was added to 10 *μ*L of transformed protoplast suspension. After 10 min incubation, protoplasts were carefully washed 3 times with W5 solution (2 mm MES pH 5.7, 154 mm NaCl, 125 mm CaCl_2_, and 5 mm KCl). Protoplasts were mounted and imaged using a fluorescence microscope with a 40× objective to visualize subcellular distributions.

### Transient expression in *N. benthamiana*

*A. tumefaciens* strain GV3101 harboring each construct was grown overnight in 5 mL of LB media and pelleted by centrifugation at 3,000 × *g*. The bacterial pellet was resuspended in 2.5 mL of infiltration buffer (10 mm MES pH 5.7, 10 mm MgCl_2_) with 200 *μ*m acetosyringone. The bacterial suspension was diluted to an OD_600_ of 1.0 with infiltration buffer. The bacterial suspensions were infiltrated into the abaxial side of leaves from 3-wk-old *N. benthamiana*. An equal volume of cell suspension harboring RNA-silencing inhibitor P19 was co-infiltrated. Approximately 50 h after infiltration, leaf punches were mounted in 1× phosphate-buffered saline and imaged using a fluorescence microscope with 20× or 63× objectives.

### Fluorescence imaging and microscopy analysis

Fluorescence microscopy was performed using a Zeiss Axio Observer 7 inverted microscope equipped with Plan-Apochromat 20×/0.8, EC Plan-Neofluar 40×/0.8, and C-Apochromat 63×/1.2 objectives, and an Axiocam 705 mono camera (Carl Zeiss). Fluorescence images were obtained using a Colibri 7 solid-state LED light source and the following Zeiss filter sets: DAPI was imaged using excitation at 385/30 nm (UV LED, 20% intensity) with emission collected through a bandpass (BP) 445/50 filter (Zeiss filter set 49). CFP was imaged using excitation at 423/44 nm (violet LED, 20% intensity) with emission collected through a BP 480/40 filter (Zeiss filter set 47). YFP was imaged using excitation at 511/44 nm (cyan LED, 15% to 20% intensity) with emission collected through a BP 535/30 filter (Zeiss filter set 46). Chlorophyll autofluorescence was detected using excitation at 555/30 nm (green LED, 10% to 15% intensity) with emission collected through a BP 640/86 filter (Zeiss filter set 121). Data acquisition was controlled using Zeiss ZEN Blue software. Images were acquired with an exposure time of 150 ms per channel and constant camera gain settings, ensuring comparable signal detection across genotypes. All images within a given experiment were captured under identical light intensity and exposure conditions to allow for direct comparison.

### Hypocotyl measurements and imaging of seedling phenotype

For hypocotyl length measurements, 4-d-old seedlings grown under 50 *μ*mol m^−2^ s^−1^ red light were scanned using a Brother MFC-L2750DW scanner, and hypocotyls were measured using NIH ImageJ software (https://imagej.net/ij/). Approximately 30 seedlings for each genotype were statistically analyzed. Box-and-whisker plots of hypocotyl measurements were drawn using Prism 10 software (GraphPad software). Images of representative seedlings were captured using an AmScope 64-LED stereomicroscope (AmScope). For imaging of the greening phenotype used in Figs. 3A and 4A, representative seedlings in the 20 *μ*mol m^−2^ s^−1^ red light condition (R20) were imaged using an AmScope 64-LED stereomicroscope (AmScope).

### RNA extraction and reverse transcription quantitative PCR

Preparation of RNA samples from seedlings of the indicated genotypes and growth conditions was performed using a Quick-RNA MiniPrep Kit with on-column DNase I treatment (Zymo Research). cDNA synthesis was performed with 1.5 *µ*g of total RNA using a Superscript IV First-Strand cDNA Synthesis Kit (Thermo Fisher Scientific) according to the manufacturer's protocol. Oligo(dT) primer and a mixture of plastidial gene-specific primers were used to analyze nuclear and plastidial genes. Quantitative PCRs (qPCRs) were performed in 96-well plates on a LightCycler 96 System (Roche) using FastStart Essential DNA Green Master (Roche) in a volume of 10 *μ*L. The *PP2A* gene was included as an internal control in the PCRs to normalize for variations in the amounts of cDNA used. Primers for reverse transcription qPCR (RT-qPCR) and cDNA synthesis are listed in Supplementary Table S2.

### Protein extraction and immunoblot analysis

Total protein was extracted from Arabidopsis seedlings grown under the indicated conditions. Plant tissues were ground in liquid nitrogen and resuspended in extraction buffer (100 mm Tris-HCl pH 7.5, 100 mm NaCl, 1% SDS, 5 mm EDTA pH 8.0, 20 mm DTT, 40 *μ*m MG132, 40 *μ*m MG115, and EDTA-free protease inhibitor cocktail) at room temperature and then centrifuged at 20,000 × *g* for 10 min. The supernatant was mixed with the same volume of 2× SDS loading buffer and boiled for 10 min. Protein extracts were separated on an SDS-PAGE mini-gel and transferred onto a PVDF membrane. The membrane was blocked in 5% nonfat milk in 1× Tris-buffered saline with Tween-20 and detected with mouse monoclonal anti-HA (H9658, Sigma-Aldrich) and anti-Actin (A0480, Sigma-Aldrich) antibodies. An anti-mouse (1706516, Bio-Rad) secondary antibody was used at a 1:5,000 dilution. The signals were detected with a chemiluminescence reaction using SuperSignal West Dura Extended Duration Chemiluminescent Substrate (Thermo Fisher Scientific).

### Quantification of stromules

Stromule frequency was calculated as the percentage of the total number of stromules per the total number of chloroplasts, following the protocol (Brunkard et al. 2016). Bar graphs were generated using Prism 10 (GraphPad Software).

## Accession numbers

Sequence data from this article can be found in the GenBank/EMBL data libraries under accession numbers: *PP2A* (At1g69960), *NCP* (At2g31840), *RCB* (At4g28590), *psbA* (AtCg00020), *rbcL* (AtCg00490), *rpoA* (AtCg00740), *rpoB* (AtCg00190), *RBCS1A* (At1g67090), *PIF1* (At2g20180), *PIF3* (AT1g09530), *PIF4* (At2g43010), and *PIF5* (At3g59060).

## Supplementary Material

kiaf289_Supplementary_Data

## Data Availability

The data underlying this article are available in the article and its online supplementary material.

## Supplementary data

The following materials are available in the online version of this article.

**Supplementary Figure S1.** Light controls alternative TSSs of *NCP*.

**Supplementary Figure S2.** Identification of *NCP* and *RCB* 5′ cDNA ends.

**Supplementary Figure S3.** Schematic illustration of the predicted domain structure of NCP-L and NCP-S isoforms.

**Supplementary Figure S4.** The cleavage site of the NCP chloroplast transit peptide is mapped between amino acids 60 and 70.

**Supplementary Figure S5.** NCP-L-CFP and NCP-S-CFP proteins show distinct subcellular localization, and the tagged protein isoforms are intact in *N. benthamiana*.

**Supplementary Figure S6.** Localization patterns of NCP-L isoform in *N. benthamiana* leaves.

**Supplementary Figure S7.** Localization patterns of NCP-S isoform with different fusion tags.

**Supplementary Figure S8.** Hypocotyl length is unaffected by *NCP-S* overexpression in etiolated seedlings.

**Supplementary Figure S9.** Levels of *NCP* transcripts in the *NCPpro:NCP-HA-His/ncp-10* plants.

**Supplementary Figure S10.** Protoplast isolation and control expression of cTP-YFP in *ncp-10* albino protoplasts.

**Supplementary Figure S11.** cTP-YFP-rpoA fusion protein localizes to chloroplast nucleoids.

**Supplementary Figure S12.**  *NCP* overexpression does not alter hypocotyl responses under red light.

**Supplementary Table S1.** Primers used in construct preparation.

**Supplementary Table S2.** Primers used in genotyping PCR, cDNA synthesis, and RT-qPCR.

**Supplementary Table S3.** Primers used for 5′ RACE-PCR and the number of PCR cycles.

## Dive Curated Terms

The following phenotypic, genotypic, and functional terms are of significance to the work described in this paper:

WHY1 Gramene: AT1G14410
WHY1 Araport: AT1G14410

UBQ10 Gramene: AT4G05320
UBQ10 Araport: AT4G05320

PP2A Gramene: AT1G69960
PP2A Araport: AT1G69960

DAPI CHEBI: CHEBI:51231

PIF3 Gramene: AT1G09530
PIF3 Araport: AT1G09530

RBCS1A Gramene: AT1G67090
RBCS1A Araport: AT1G67090

pifq Gramene: pif1 pif3 pif4 pif5
pifq Araport: pif1 pif3 pif4 pif5

NCP Gramene: AT2G31840
NCP Araport: AT2G31840

RCB Gramene: AT4G28590
RCB Araport: AT4G28590

PIF1 Gramene: AT2G20180
PIF1 Araport: AT2G20180
