# Supplementary material for: Light-regulated dual-targeting of NUCLEAR CONTROL OF PEP ACTIVITY establishes photomorphogenesis via interorganellar communication
Source: Plant Physiol. 2025 Jun 30;199(1):kiaf289. doi: 10.1093/plphys/kiaf289 (PMC12412214; doi:10.1093/plphys/kiaf289)
Supplement: kiaf289_Supplementary_Data [file kiaf289_supplementary_data.pdf]

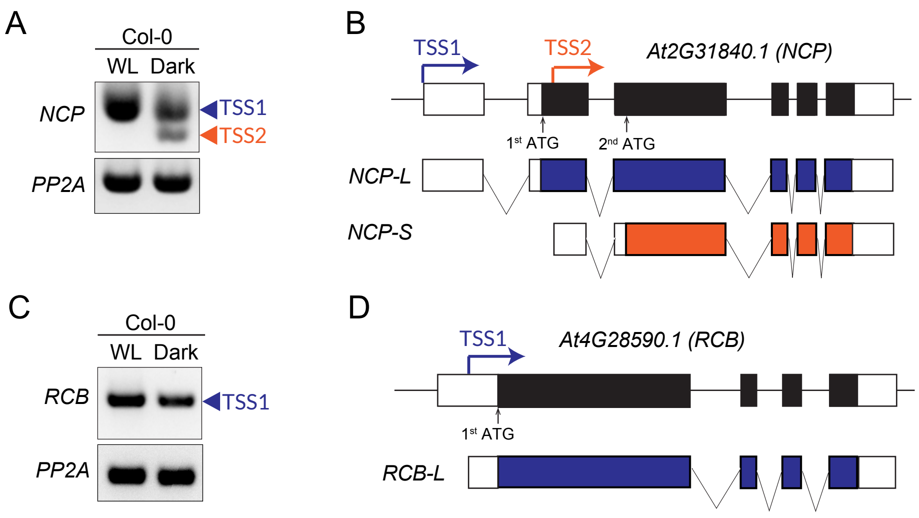

Supplemental Figure S1. Light controls alternative transcription start sites of *NCP*.

(A) Identification of *NCP* transcripts with different transcription start sites (TSSs). Wild-type (Col-0) plants were grown on MS agar plates either under 80 μmol m^{-2} s^{-1} white light (WL) or in darkness for 4 d before extracting total RNA from whole seedlings. Two specific bands were detected by 5' RACE-PCR. The specific bands were confirmed by Sanger sequencing, and the transcription start sites were designated as TSS1 (blue arrowhead) and TSS2 (orange arrowhead). *Protein phosphatase 2A* (*PP2A*) was used as an internal control.

(B) Schematic representation of *NCP* transcripts with different transcription start sites. The longer and shorter *NCP* transcripts correspond to *NCP-L* and *NCP-S*, respectively. Predicted start codons associated with each transcription start sites are depicted below. Untranslated regions are shown as white boxes, exons as dark boxes, and introns as horizontal lines.

(C and D) 5' RACE analysis of the *RCB* gene. Seedling growth and RNA extraction were performed as described in (A). The transcription start site identified was designated as TSS1 (blue arrowhead) (C). *PP2A* was used as an internal control. The longer *RCB* transcript cloned by 5' RACE was represented as *RCB-L* (D).
The TSS1 of *RCB-L* was verified to be upstream of the annotated TSS (TAIR).

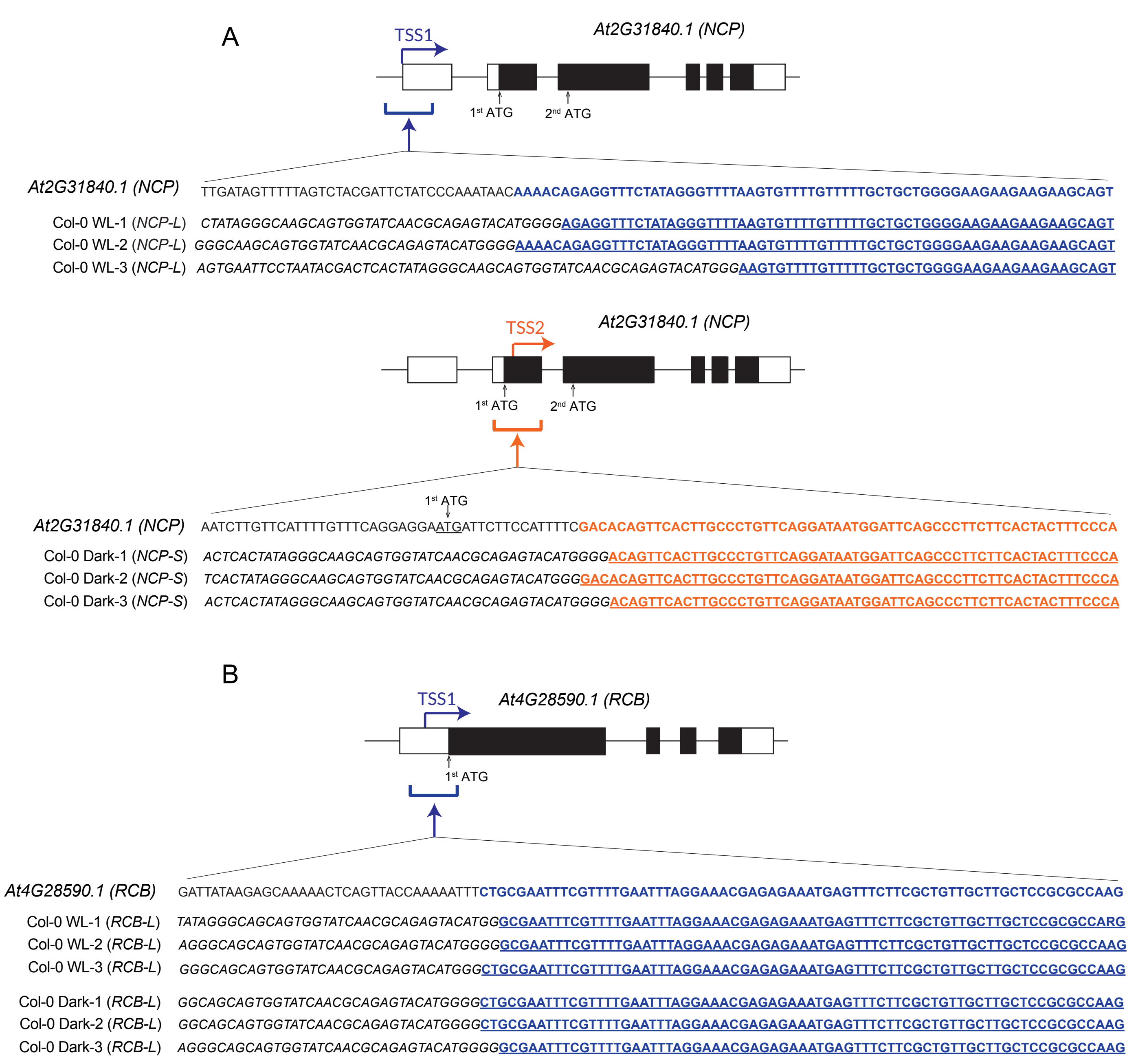

Supplemental Figure S2. Identification of *NCP* and *RCB* 5' cDNA ends.

(A) Identification of *NCP* 5' cDNA ends with different transcription start sites (TSSs). Each *NCP* transcript amplified from 5' RACE-PCR was subcloned into a linearized pRACE vector (Clontech). At least three independent *NCP* 5' RACE clones for each reaction (WL and Dark) were sequenced. Sequences were aligned with the annotated *NCP* sequence (TAIR). The underlined blue characters indicate matched DNA sequences from *NCP-L* transcript and the underlined orange characters denote aligned sequences from *NCP-S*. The *NCP-S* transcript started with TSS2 just after 1st ATG in the coding sequence. The italicized black DNA sequences resulted from SMARTer 5' RACE cDNA synthesis (Clontech). Black boxes and open boxes represent coding regions and untranslated regions (UTRs), respectively. Black line indicate non-coding regions.

(B) Identification of *RCB* 5' cDNA end. Preparation of 5' RACE clones was described as in (A). Three independent 5' RACE clones for each reaction were sequenced and displayed. Blue bracket indicates the region sequence aligned. The underlined blue characters denote matched DNA sequences from *RCB-L* transcript with annotated *RCB* sequence. The italicized black DNA sequences resulted from SMARTer 5' RACE cDNA synthesis. Black boxes, open boxes, and black lines represent coding regions, UTRs, and non-coding regions, respectively.

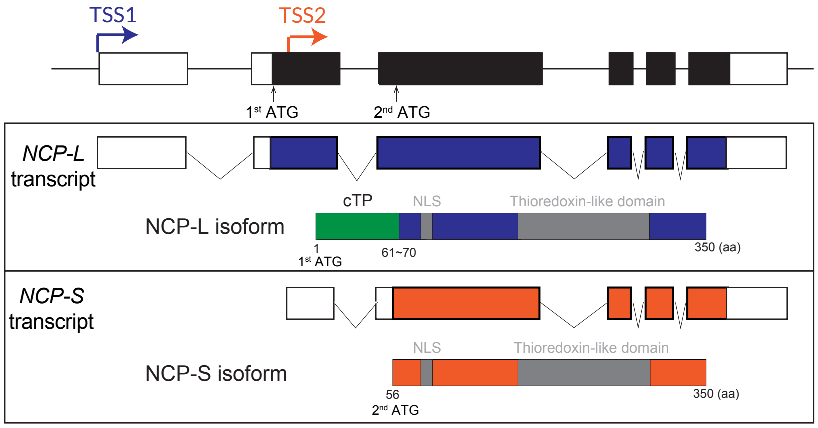

Supplemental Figure S3. Schematic illustration of the predicted domain structure of NCP-L and NCP-S isoforms. Black boxes, open boxes, and black lines represent coding regions, UTRs, and non-coding regions, respectively. The chloroplast-targeting transit peptide (cTP) of the NCP-L isoform is indicated by a green box. The putative nuclear localization signal (NLS) and thioredoxin-like domains are indicated by gray boxes.

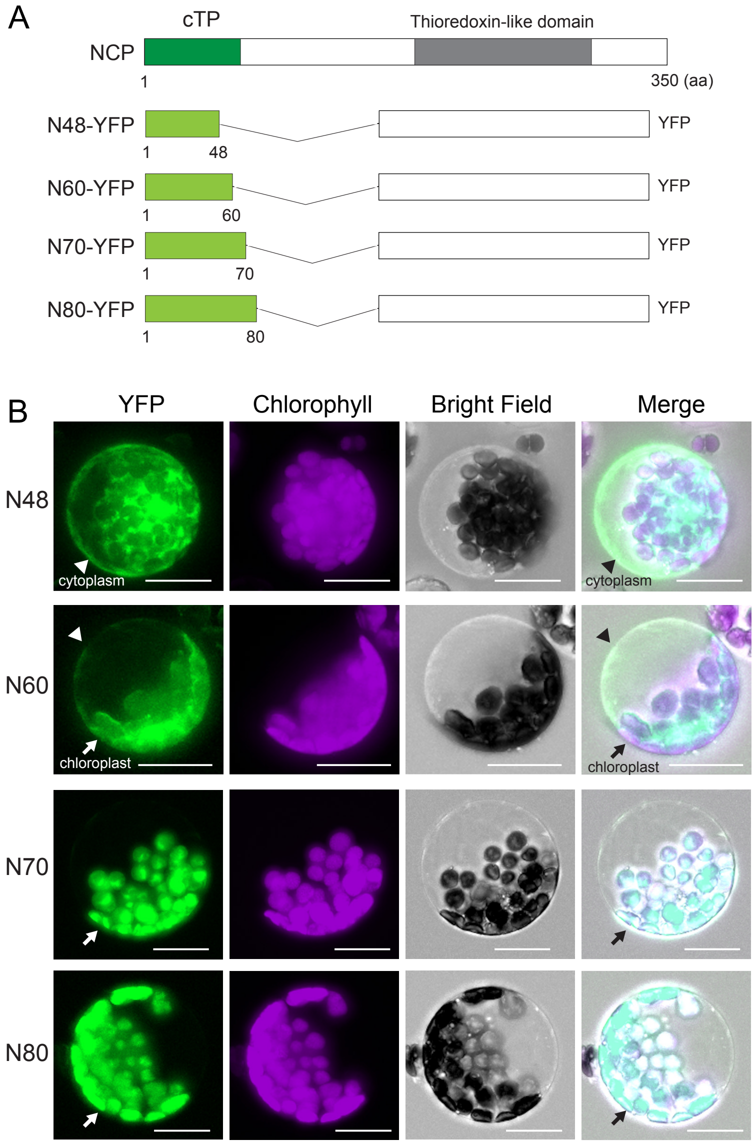

Supplemental Figure S4. The cleavage site of the NCP chloroplast transit peptide is mapped between amino acids 60 and 70.

(A) Schematic illustration of the domain structure of NCP and a series of N-terminal fragments of NCP fused to YFP. cTP, chloroplast-targeting transit peptide.

(B) Subcellular localization patterns of N-terminal NCP fragments fused to YFP in Arabidopsis protoplasts. Each construct was expressed transiently and visualized by bright field and fluorescence microscopy. Thick arrows indicate chloroplasts, and filled arrowheads denote cytoplasmic signals. Chlorophyll, autofluorescence. Scale bars, 20 μm.

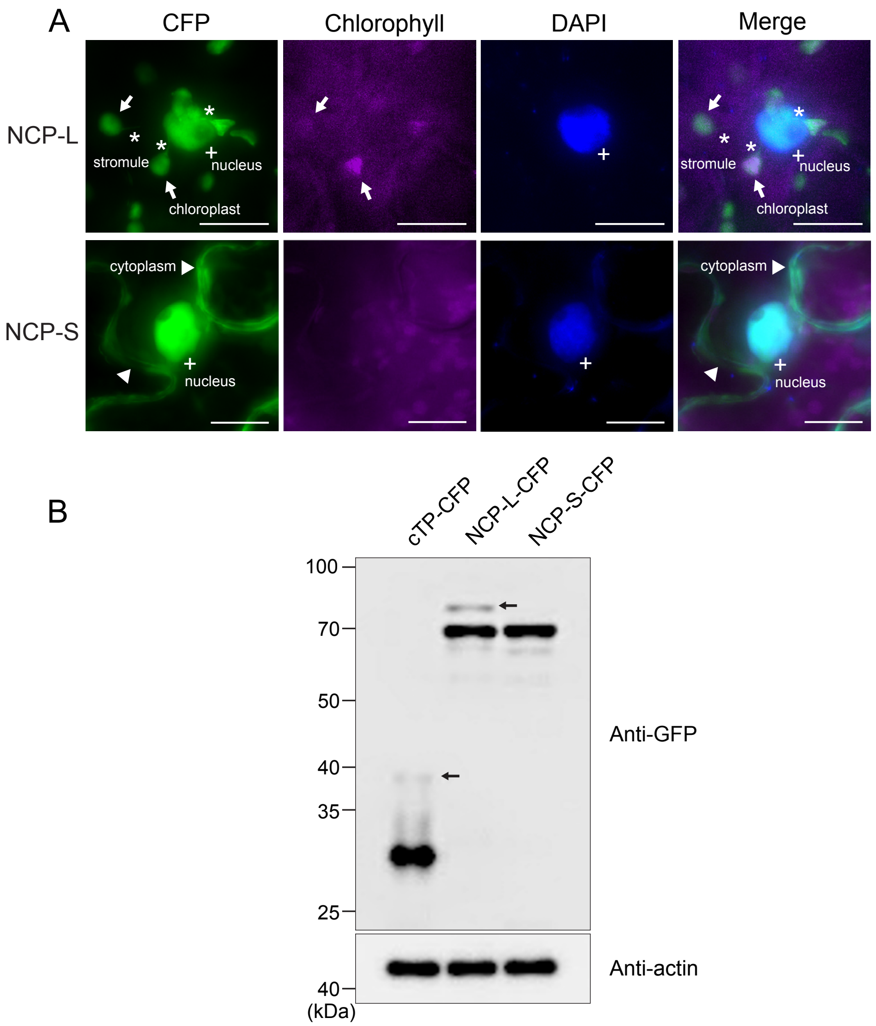

Supplemental Figure S5. NCP-L-CFP and NCP-S-CFP proteins show distinct subcellular localization, and the tagged protein isoforms are intact in *N. benthamiana*.

(A) Distinct localization of NCP-L-CFP and NCP-S-CFP proteins. The *UBQ10pro:NCP-L-CFP* (upper panel) and *UBQ10pro:NCP-S-CFP* (lower panel) fusion constructs were transiently expressed in *N. benthamiana* leaves. Fluorescence microscopy images of epidermal cells were visualized. DAPI was used for staining nuclei. Plus signs (+) and asterisks indicate the nucleus and stromules, respectively. Arrows indicate chloroplasts, and arrowheads denote cytoplasmic signals. Chlorophyll, autofluorescence. Scale bars, 20 μm.

(B) The tagged NCP-L (NCPm) and NCP-S protein isoforms are intact. For the control construct (*cTP-CFP*), the coding sequence of the N-terminal chloroplast transit peptide (cTP) of *RBCS1A* was transcriptionally fused to the 5' end of the CFP-coding sequence under the control of *UBQ10* promoter. The same *NCP-L-CFP* and *NCP-S-CFP* fusion constructs used in (A) were expressed in *N. benthamiana* leaves. An anti-GFP antibody was used to detect NCP-L-CFP and NCP-S-CFP proteins. Actin was used as a loading control. Arrows indicate pre-mature forms of the proteins.

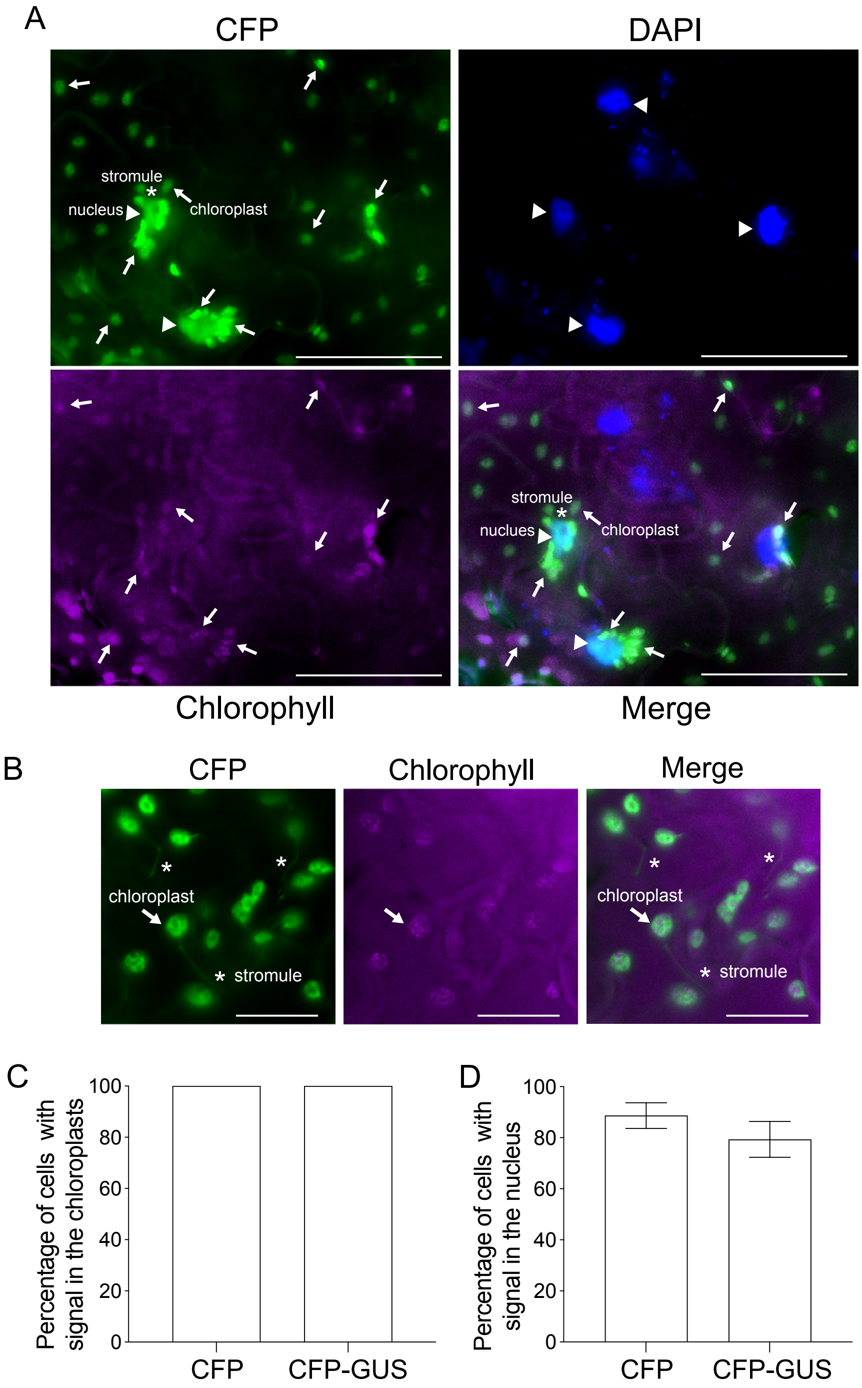

Supplemental Figure S6. Localization patterns of the NCP-L isoform in *N. benthamiana* leaves.

(A) Overall expression patterns of the NCP-L isoform. The *UBQ10pro:NCP-L-CFP* fusion construct was transiently expressed in *N. benthamiana* leaves. Fluorescence microscopy images of epidermal cells were visualized. NCP-L-CFP signals were mostly detected in chloroplasts (arrows) and often observed in the nucleus (arrowheads) when chloroplasts surround them. Asterisks denote stromules. DAPI was used for staining nuclei. Chlorophyll, autofluorescence. Scale bars, 50 μm.

(B) Expression of the NCP-L isoform in stromules. The *UBQ10pro:NCP-L-CFP* construct was transiently expressed in *N. benthamiana* leaves and visualized by fluorescence microscopy. Arrows and asterisks denote chloroplast and stromules, respectively. Chlorophyll, autofluorescence. Scale bars, 20 μm.

(C and D) Quantification of NCP-L localization. The *UBQ10pro:NCP-L-CFP* and *UBQ10pro:NCP-L-CFP*-GUS constructs were transiently expressed in *N. benthamiana* leaves. The percentage of cells with CFP or CFP-GUS signals in the chloroplast (C) and nucleus (D) was calculated. Three independent measurements, each consisting of 60 cells, were averaged.
Error bars indicate SD.

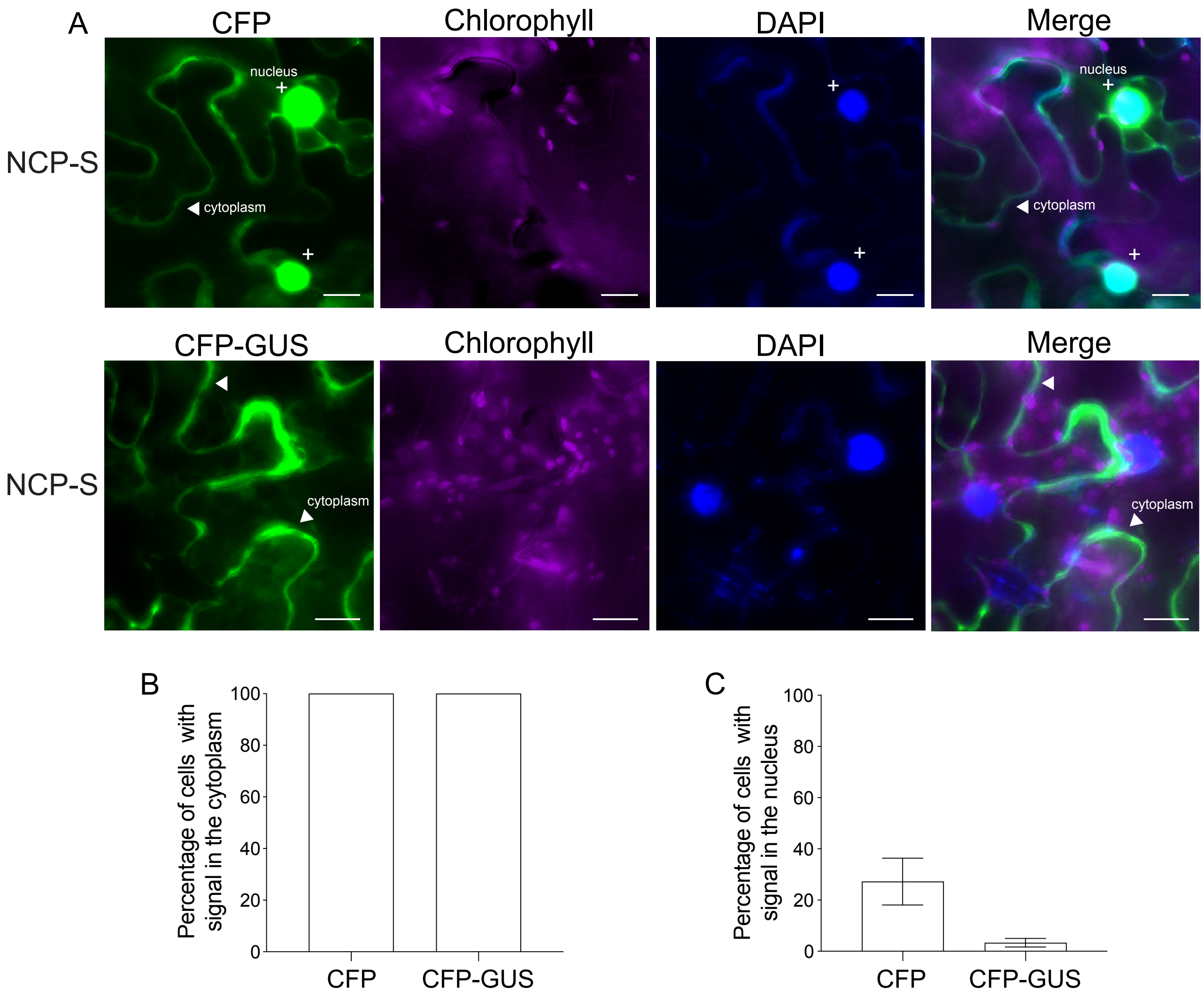

Supplemental Figure S7. Localization patterns of the NCP-S isoform with different fusion tags.

(A) Subcellular localization patterns of NCP-S-CFP and NCP-S-CFP-GUS proteins. The *UBQ10pro:NCP-S-CFP* (upper panel) and *UBQ10pro:NCP-S-CFP-GUS* (lower panel) fusion constructs were transiently expressed in *N. benthamiana* leaves. Fluorescence microscopy images of epidermal cells were visualized. DAPI was used for staining nuclei. Plus signs (+) and arrowheads denote nuclear and cytoplasmic signals, respectively. Chlorophyll, autofluorescence. Scale bars, 20 μm.

(B and C) Quantification of NCP-S localization. The *UBQ10pro:NCP-S-CFP* and *UBQ10pro:NCP-S-CFP*-GUS constructs were transiently expressed in *N. benthamiana* leaves. The percentage of cells with CFP or CFP-GUS signals in the cytoplasm (B) and nucleus (C) was calculated. Three biological replicates, each consisting of 50 cells, were averaged. Error bars indicate SD.

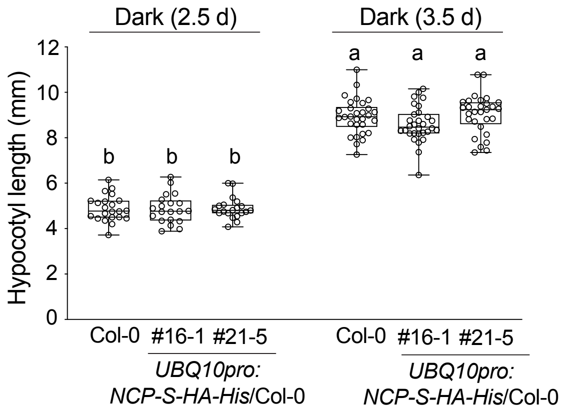

Supplemental Figure S8. Hypocotyl length is unaffected by *NCP-S* overexpression in etiolated seedlings.

Box-and-whisker plots showing hypocotyl length of 2.5-d-old and 3.5-d-old Col-0 and *UBQ10pro:NCP-S-HA-His*/Col-0 seedlings grown in darkness. Boxes indicate the 25th to 75th percentiles with median values shown as horizontal lines; whisker extend to the minimum and maximum values. No outliers were detected. Sample size (n): Col-0 (22 dark, 30 light), NCP-S-HA-His #16-1 (20 dark, 30 light), NCP-S-HA-His #21-5 (20 dark, 29 light). Different letters represent significant differences ($P < 0.001$, one-way ANOVA with post-hoc Tukey's HSD test).

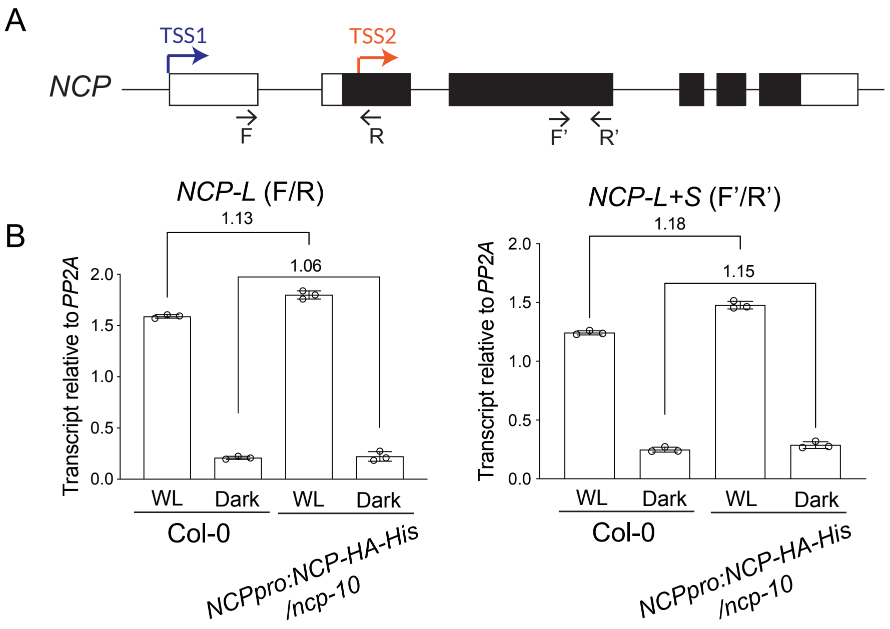

Supplemental Figure S9. Levels of *NCP* transcripts in the *NCPpro:NCP-HA-His/ncp-10* plants.

(A) Schematic showing primer positions (indicated by black arrows) used to amplify *NCP* transcripts. Untranslated regions are shown as white boxes, and exons are shown as dark boxes.

(B) RT-qPCR analysis of *NCP* transcript levels originating from different transcription start sites. Col-0 and *NCPpro:NCP-HA-His/ncp-10* seedlings were grown under white light (WL) or in the dark for 4 d before harvesting whole seedlings for RNA extraction. Transcript levels were normalized to those of *PP2A*. Error bars represent SD (n = 3). Fold changes between Col-0 and *NCPpro:NCP-HA-His/ncp-10* samples are indicated.

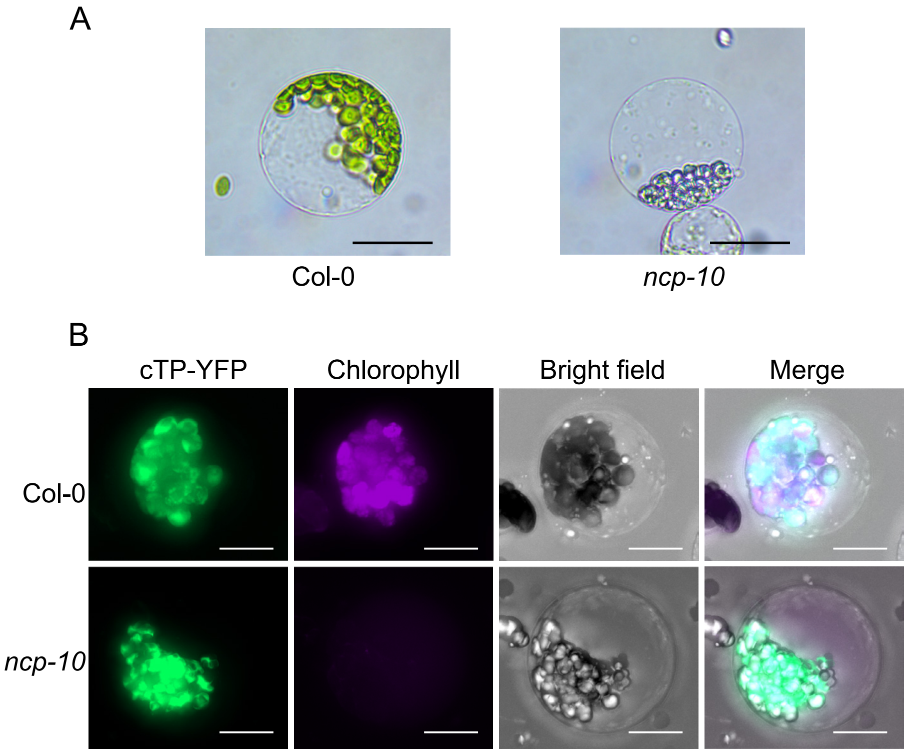

Supplemental Figure S10. Protoplast isolation and control expression of cTP-YFP in *ncp-10* albino protoplasts.

(A) Protoplasts isolated from *ncp-10* mutant. Seedlings of Col-0 wild type and *ncp-10* knockout mutant were grown for 3 weeks on agar medium supplemented with 3% sucrose. The panels show bright field images, which indicate the absence of mature chloroplasts in *ncp-10* protoplasts. Scale bars, 20 μm.

(B) Expression of cTP-YFP in *ncp-10* protoplasts. The coding sequence of the N-terminal chloroplast transit peptide (cTP) of *RBCS1A* was fused to the 5’ end of the YFP-coding sequence under the control of the CaMV 35S promoter. The cTP-YFP construct was transiently expressed in Arabidopsis protoplasts and visualized by fluorescence microscopy. Scale bars, 10 μm.

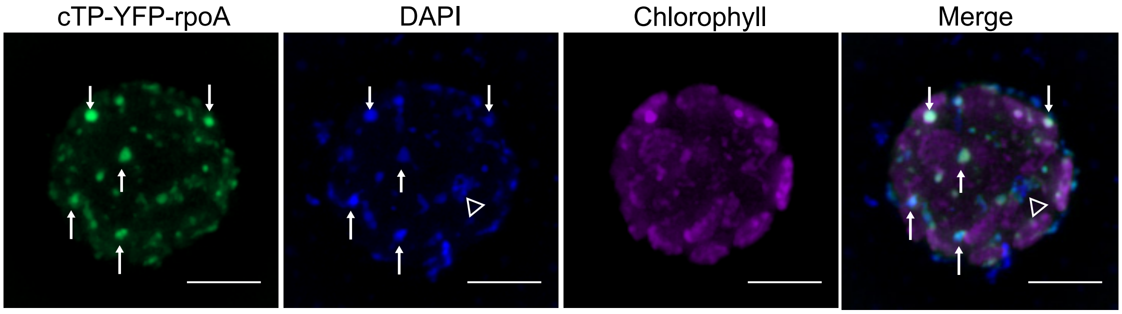

Supplemental Figure S11. cTP-YFP-rpoA fusion protein localizes to chloroplast nucleoids.

DAPI staining of Col-0 protoplasts expressing cTP-YFP-rpoA. Protoplasts were stained with DAPI and observed by fluorescence microscopy. Arrows indicate nucleoids, and the empty arrowhead denotes the nucleus. Scale bars, 10 μm.

.

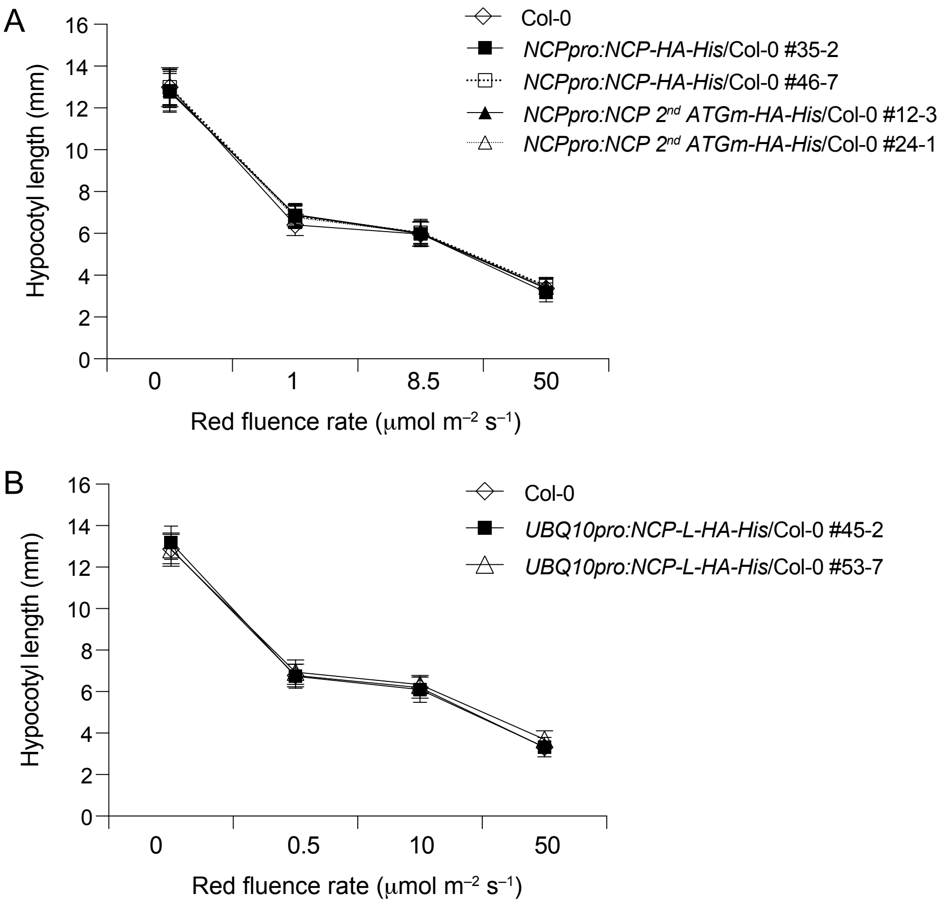

Supplemental Figure S12. *NCP* overexpression does not alter hypocotyl responses under red light.

(A) Red (R) light fluence curves showing hypocotyl elongation responses of 4-day-old Col-0, *NCPpro:NCP-HA-His*/Col-0, and *NCPpro:NCP 2nd ATGm-HA-His*/Col-0 seedlings grown in the dark or a series of red light intensities. Error bars represents SD. Sample sizes for dark, R1, R8.5, R50 were: Col-0 (33, 30,31, 30), NCP #35-2 (30, 30, 29, 30), NCP #46-7 (31, 29, 30, 30), *NCP 2nd ATGm #12-3* (34, 30, 31, 30), and *NCP 2nd ATGm #24-1* (36, 31, 31, 30).

(B) Red light fluence curves for Col-0, *UBQ10pro:NCP-L-HA-His*/Col-0 measured by growing seedlings for 4 days in darkness or a series of red light intensities. Error bars represent SD. Sample sizes for dark, R0.5, R10, R50 were: Col-0 (32, 32, 30, 30), *NCP-L #45-2* (30, 31, 31, 30), and *NCP-L #53-7* (30, 31, 30, 30).

Supplemental Table S1. Primers used in construct preparation.

Construct	Primer	Sequences (5'- 3')	Usage
UBQ10pro:NCP-L-CFP	NCP-L CDS_fwd	CAGCGAGCTCGGTACCCGGGATGATTCTTCCATTTTCGAC	Subcloning
UBQ10pro:NCP-L(S)-CFP	NCP CDS_rev	CTTTACTCATATAATTCACACTTACATCGAC	Subcloning
UBQ10pro:NCP-L(S)-CFP	CFP_fwd	TGTGAATTATATGAGTAAAGGAGAAGAAC	Subcloning
UBQ10pro:NCP-L(S)-CFP	CFP_rev	GAAAGCTCTGCATGCCTGCATCACTTGTCATCGTCATC	Subcloning
UBQ10pro:NCP-S-CFP	NCP-S CDS_fwd	CAGCGAGCTCGGTACCCGGGATGAGGTCGAGGAGAAATG	Subcloning
UBQ10pro:NCP-L(S)-CFP-GUS	CFP_rev	GACGTAACATCTTGTCATCGTCATCCTTG	Subcloning
UBQ10pro:NCP-L(S)-CFP-GUS	GUS_fwd	CGATGACAAGATGTTACGTCCTGTAGAAAC	Subcloning
UBQ10pro:NCP-L(S)-CFP-GUS	GUS_rev	GAAAGCTCTGCATGCCTGCATCATTGTTTGCCTCCCTG	Subcloning
UBQ10pro:NCPΔ48-CFP-GUS	NCPm_fwd	AACAGAGCTCGGTACCCGGGATGTCCTCCTCGAAGTGG	Subcloning
UBQ10pro:cTP-NCPΔ48-CFP-GUS	cTP_fwd	AACAGAGCTCGGTACCCGGGATGGCTTCCTCTATGCTC	Subcloning
UBQ10pro:cTP-NCPΔ48-CFP-GUS	cTP_rev	TCGAGGAGGAAAGCTTAAGGTCAGGAAG	Subcloning
UBQ10pro:cTP-NCPΔ48-CFP-GUS	NCPm_fwd	CCTTAAGCTTTCCTCCTCGAAGTGGAATG	Subcloning
UBQ10pro:cTP-CFP	cTP_rev	CTTTACTCATAAGCTTAAGGTCAGGAAG	Subcloning
UBQ10pro:cTP-CFP	CFP_fwd	CCTTAAGCTTATGAGTAAAGGAGAAGAAC	Subcloning
NCPpro:NCP-HA-His	gNCP_fwd	ACAGCTATGACATGATTACGTTATCACATCCATTCACATTTG	Subcloning
NCPpro:NCP-HA-His	gNCP_rev	CCCGGGTACCATTCACACTTACATCGAC	Subcloning
NCPpro:NCP-HA-His	HA-His_fwd	AAGTGTGAATGGTACCCGGGGATCCTCTAG	Subcloning
NCPpro:NCP-HA-His	HA-His_rev	CCTGCAGGTCGACTCTAGAGTCAGTGATGGTGATGGTGATG	Subcloning
NCPpro:NCP 2nd ATGm-*HA-His*	NCP 2nd ATGm _fwd	AAGTGGAATGTTTTGAGGTCGAGGAGA	Subcloning
NCPpro:NCP 2nd ATGm-*HA-His*	NCP 2nd ATGm _rev	TCTCCTCGACCTCAAAACATTCCACTT	Subcloning
UBQ10-NCP-L-HA-His	NCP-L CDS_fwd	CAGCGAGCTCGGTACCCGGGATGATTCTTCCATTTTCGAC	Subcloning
UBQ10-NCP-L(S)-HA-His	NCP CDS_rev	CCCGGGTACCATTCACACTTACATCGAC	Subcloning
UBQ10-NCP-L(S)-HA-His	HA-His_fwd	AAGTGTGAATGGTACCCGGGGATCCTCTAG	Subcloning
UBQ10-NCP-L(S)-HA-His	HA-His_rev	GAAAGCTCTGCATGCCTGCATCAGTGATGGTGATGGTGATG	Subcloning
UBQ10-NCP-S-HA-His	NCP-S CDS_fwd	CAGCGAGCTCGGTACCCGGGATGAGGTCGAGGAGAAATG	Subcloning
UBQ10pro:NCPΔ48-HA-His	NCPΔ48_fwd	AACAGAGCTCGGTACCCGGGATGTCCTCCTCGAAGTGG	Subcloning
35Spro:N48aa-YFP	NCP_fwd	TGGAGAGAACACGGGGGACTATGATTCTTCCATTTTCGAC	Subcloning

35Spro:N48aa-YFP	NCP 1-48aa_rev	TGCTCACCATGGCAGTCTTCACCGAACC	Subcloning
35Spro:N48aa-YFP	YFP_fwd	GAAGACTGCCATGGTGAGCAAGGGCGAG	Subcloning
35Spro:N60aa-YFP	NCP 1-60aa_rev	TGCTCACCATTCTCCTCGACCTCATAAC	Subcloning
35Spro:N60aa-YFP	YFP_fwd	GTCGAGGAGAATGGTGAGCAAGGGCGAG	Subcloning
35Spro:N70aa-YFP	NCP 1-70aa_rev	TGCTCACCATTTTATCAACTAACCCAAAAGC	Subcloning
35Spro:N70aa-YFP	YFP_fwd	AGTTGATAAAATGGTGAGCAAGGGCGAG	Subcloning
35Spro:N80aa-YFP	NCP 1-80aa_rev	TGCTCACCATTTCTTTCTTTCTCCATACC	Subcloning
35Spro:N80aa-YFP	YFP_fwd	AAAGAAAGAAATGGTGAGCAAGGGCGAG	Subcloning
35Spro:cTP-YFP-rpoA	cTP-YFP_rev	CTCGAACCATCTTGTACAGCTCGTCCATG	Subcloning
35Spro:cTP-YFP-rpoA	rpoA_fwd	GCTGTACAAGATGGTTCGAGAGAAAGTC	Subcloning
35Spro:cTP-YFP-rpoA	rpoA_rev	ATCGGGGAAATTCGAGGTACCTATTTTTTTTCTAGAATGTCTAATATC	Subcloning
35Spro:cTP-YFP-rpoB	cTP-YFP_rev	CCCCAAGCATCTTGTACAGCTCGTCCATG	Subcloning
35Spro:cTP-YFP-rpoB	rpoB_fwd	GCTGTACAAGATGCTTGGGGATGAAAAAG	Subcloning
35Spro:cTP-YFP-rpoB	rpoB_rev	ATCGGGGAAATTCGAGGTACCTAAACTTCCTTCCTATTAATCTG	Subcloning
pCMX-PL2-NCP_FL-HA-His	NCP_FL_fwd	CCTCGAGAAGCTTGATATCGATGATTCTTCCATTTTCGAC	Subcloning
pCMX-PL2-NCP_FL-HA-His	HA-His_rev	GCTACTAGCTAGCTGGCCAGTCAGTGATGGTGATGGTGATG	Subcloning
pCMX-PL2-NCPΔ55-HA-His	NCPΔ55_fwd	CCTCGAGAAGCTTGATATCGATGAGGTCGAGGAGAAATG	Subcloning
pCMX-PL2-NCPΔ72-HA-His	NCPΔ72_fwd	CCTCGAGAAGCTTGATATCGATGAAGAAGGTATGGAGAAAG	Subcloning

The PCR primers were designed using NEBuilder software (version 2.10.1, https://nebuilder.neb.com/#!/) so that they have calculated melting temperatures in the range of 55−65°C. fwd, forward primer; rev, reverse primer.

Supplemental Table S2. Primers used in genotyping PCR, cDNA synthesis, and RT-qPCR.

Primer	Sequences (5'-3')	Usage
*ncp-10*_LP	AAGGGAGAAGAGAGCACGTT	Genotyping
*ncp-10*_RP	GTAGAGAGACGGGAATGGAGG	Genotyping
*ncp-10*_LB	ATAATAACGCTGCGGACATCTACATTTT	Genotyping
NCP-S-HA-His transgene_fwd	GTAGAGAGACGGGAATGGAGG	Genotyping
NCP-S-HA-His transgene_rev	GAAAGCTCTGCATGCCTGCATCAGTGATGGTGATGGTGATG	Genotyping
*psbA*_cDNA	TAGATGGAGCCTCAACAGCAGCTA	cDNA synthesis
*rbcL*_cDNA	CTTCACAAGCAGCAGCTAGTTCAGG	cDNA synthesis
*psbA*_fwd	ACATTTCTTCTTAGCGGCTT	RT-qPCR
*psbA*_rev	CGTCCTTGACTATCAACTACTGA	RT-qPCR
*rbcL*_fwd	GGAGATGATTCTGTACTACAAT	RT-qPCR
*rbcL*_rev	GTCCCTCATTACGAGCTTGTAC	RT-qPCR
NCP-L(S)-HA-His transgene_fwd	CTTGTTTGAATGGTGTCGTGAAT	RT-qPCR
NCP-L(S)-HA-His transgene_rev	GTTGGTGTAGGTGTTGGAGTAG	RT-qPCR
NCP-L_fwd	GCAAATACAGTTCTGTTG	RT-qPCR
NCP-L_rev	GGGAAAGTAGTGAAGAAG	RT-qPCR
NCP-L+S_fwd	AGGAGATGATAGTGAGAAGGAGA	RT-qPCR
NCP-L+S_rev	CTTTGAACACCGCCTCTTCC	RT-qPCR

The PCR primers were designed using Primer3 software (version 4.1.0, https://primer3.ut.ee/) in a way that they have calculated melting temperatures in a range of 50-60°C. fwd, forward primer; rev, reverse primer.

Supplemental Table S3. Primers used for 5' RACE-PCR and the number of PCR cycles.

Primer	Sequences (5'-3')	PCR cycles
Universal Primer A Mix (UPM)	CTAATACGACTCACTATAGGGCAAGCAGTGGTATCAACGCAGAGT	30 cycles
NCP 5' RACE_GSP	GATTACGCCAAGCTTACTTGAATCGCCTTCTCTAGTTCTTCCC	30 cycles (with UPM)
Universal Primer Short (UPS)	CTAATACGACTCACTATAGGGC	25 cycles
NCP 5' RACE_NGSP	GATTACGCCAAGCTTGTCTTCCTCCATTCCCGTCTCTCTACTG	25 cycles (with UPS)
RCB 5' RACE_GSP	GATTACGCCAAGCTTCTCCCTGTACAGAATCCGGT	30 cycles (with UPM)
RCB 5' RACE_NGSP	GATTACGCCAAGCTTGATTTACGCGGGACCTTTGT	20 cycles (with UPS)
*PP2A*_fwd	TGCCCCAGATGTGCTAAAGA	25 cycles
*PP2A*_rev	GCTGCTATCCGAACTTCTGC	25 cycles

GSP, gene-specific primer; NGSP, nested gene-specific primer; fwd, forward primer; rev, reverse primer.
